# Lactate Regulates Metabolic and Pro-inflammatory Circuits in Control of T Cell Migration and Effector Functions

**DOI:** 10.1371/journal.pbio.1002202

**Published:** 2015-07-16

**Authors:** Robert Haas, Joanne Smith, Vidalba Rocher-Ros, Suchita Nadkarni, Trinidad Montero-Melendez, Fulvio D’Acquisto, Elliot J. Bland, Michele Bombardieri, Costantino Pitzalis, Mauro Perretti, Federica M. Marelli-Berg, Claudio Mauro

**Affiliations:** 1 William Harvey Research Institute, Barts and The London School of Medicine and Dentistry, London, United Kingdom; 2 Queen Mary Innovation Ltd, Queen Mary University of London, London, United Kingdom; National Jewish Medical and Research Center/Howard Hughes Medical Institute, UNITED STATES

## Abstract

Lactate has long been considered a “waste” by-product of cell metabolism, and it accumulates at sites of inflammation. Recent findings have identified lactate as an active metabolite in cell signalling, although its effects on immune cells during inflammation are largely unexplored. Here we ask whether lactate is responsible for T cells remaining entrapped in inflammatory sites, where they perpetuate the chronic inflammatory process. We show that lactate accumulates in the synovia of rheumatoid arthritis patients. Extracellular sodium lactate and lactic acid inhibit the motility of CD4^+^ and CD8^+^ T cells, respectively. This selective control of T cell motility is mediated via subtype-specific transporters (Slc5a12 and Slc16a1) that we find selectively expressed by CD4^+^ and CD8^+^ subsets, respectively. We further show both in vitro and in vivo that the sodium lactate-mediated inhibition of CD4^+^ T cell motility is due to an interference with glycolysis activated upon engagement of the chemokine receptor CXCR3 with the chemokine CXCL10. In contrast, we find the lactic acid effect on CD8^+^ T cell motility to be independent of glycolysis control. In CD4^+^ T helper cells, sodium lactate also induces a switch towards the Th17 subset that produces large amounts of the proinflammatory cytokine IL-17, whereas in CD8^+^ T cells, lactic acid causes the loss of their cytolytic function. We further show that the expression of lactate transporters correlates with the clinical T cell score in the synovia of rheumatoid arthritis patients. Finally, pharmacological or antibody-mediated blockade of subtype-specific lactate transporters on T cells results in their release from the inflammatory site in an in vivo model of peritonitis. By establishing a novel role of lactate in control of proinflammatory T cell motility and effector functions, our findings provide a potential molecular mechanism for T cell entrapment and functional changes in inflammatory sites that drive chronic inflammation and offer targeted therapeutic interventions for the treatment of chronic inflammatory disorders.

## Abstract 2

High levels of lactate that accumulate in chronic inflammatory sites can trigger unfavorable responses in infiltrating T cells; reducing T cells' sensitivity to lactate might offer therapeutic solutions to chronic inflammatory disorders.

## Author Summary

Acidity is a feature of inflammatory sites such as arthritic synovia, atherosclerotic plaques, and tumor microenvironments and results in part from the accumulation of lactate as a product of glycolysis under hypoxic conditions. Recently it has emerged that lactate may be more than just a bystander and might act to modulate the immune-inflammatory response. Here we report just such activity: lactate inhibits T cell motility by interfering with glycolysis that is required for T cells to migrate, it causes T cells to produce higher amounts of the proinflammatory cytokine IL-17, and it triggers loss of cytolytic activity. These phenomena are hallmark features of T cells in chronic inflammatory infiltrates. The functional changes depend on the expression of specific lactate transporters by different subsets of T cells, namely the sodium lactate transporter Slc5a12 in CD4^+^ T cells and the lactic acid transporter Slc16a1 in CD8^+^ T cells. We propose that T cells entering inflammatory sites sense high concentrations of lactate via their specific transporters. Loss of motility leads to their entrapment at the site, where through their increased production of inflammatory cytokines yet decreased cytolytic capacity, they add detrimentally to chronic inflammation. Targeting lactate transporters and/or metabolic pathways on T cells could deliver novel, invaluable therapeutics for the treatment of widespread chronic inflammatory disorders.

## Introduction

Recent studies have shed light on the interconnection between metabolism and immunity in multicellular organisms and their functional coordination for an effective establishment and resolution of immune responses. Imbalance of this delicate signaling network might lead to nonresolving inflammation and consequently to the development of chronic inflammatory disease (CID) [1].

T cells play a major role in the inflammatory process via both their cytolytic activities and the production of pro- and anti-inflammatory cytokines, which regulate immune responses. Upon antigen recognition by the T cell receptor (TCR), downstream signaling events in naïve T cells lead to activation, proliferation and differentiation into effector T cells. To maintain adequate supply of macromolecules (e.g., amino acids, nucleotides, and fatty acids) during growth, T cells undergo a metabolic switch from oxidative phosphorylation to aerobic glycolysis that is driven by signaling events generated by the TCR and the costimulatory molecule CD28 [2,3]. The metabolic machinery is also likely to directly affect T cell migratory events, as T lymphocytes continuously recirculate between different microenvironments (e.g., blood, lymphoid tissues, and peripheral tissues), which might in turn modulate T cell metabolism. In these “milieus”, they are exposed to different nutrient availability and oxygen (O_2_) tension and must adapt their metabolic pathways to effectively mediate immune responses. The direct effect of metabolism on the trafficking ability of T cells, however, is yet to be investigated [4].

A metabolic switch to aerobic glycolysis comprises the up-regulation of glycolytic enzymes and glucose transporters to the membrane, leading to an increase in glycolytic flux and the concomitant production of lactate [4]. This carbon molecule has long been considered a metabolic “waste” by-product of highly proliferating cells. Recently, this view has been challenged by the observation that lactate can act as a signaling molecule and provide a “danger” signal [5,6]. Findings that endothelial cells and human cytotoxic T lymphocyte (CTL) can take up extracellular lactate [7,8], that lactate inhibits phosphofructokinase (Pfk) [9], and that exposure to lactate leads to a dramatic change in gene expression in L6 muscle cells [10] have shed light on the complexity and scope of lactate as an active metabolite driving signaling pathways that modulate cell functions.

Proinflammatory chemokines such as chemokine (C-X-C motif) ligand (CXCL)10 are produced in response to inflammatory stimuli and attract effector T cells to the site of inflammation to fulfill their effector functions [11]. Inflammatory sites, however, are “harsh” microenvironments enriched with factors released by cellular components of the inflammatory infiltrates and the injured tissue itself that might affect the behavior of effector T cells and influence the outcome of the immune response [12]. A key feature of the inflammatory microenvironment is the accumulation of lactate, a by-product of the glycolytic pathway. Depending on the pH, lactate exists as the protonated acidic form (lactic acid) in a low pH environment or as sodium salt (sodium lactate) at basic pH [13]. In physiological conditions (pH 7.2), most of the lactate is deprotonated and is present in the negatively charged, biologically active form as lactate anion [13].

In this study, we have investigated, both in vitro and in vivo, the role of lactate in the regulation of metabolic and inflammatory circuits in control of T cell migration and functions and its relevance to the physiopathology of CID.

## Results

### Lactate Inhibits Activated T Cell Motility

**Fig 1:**
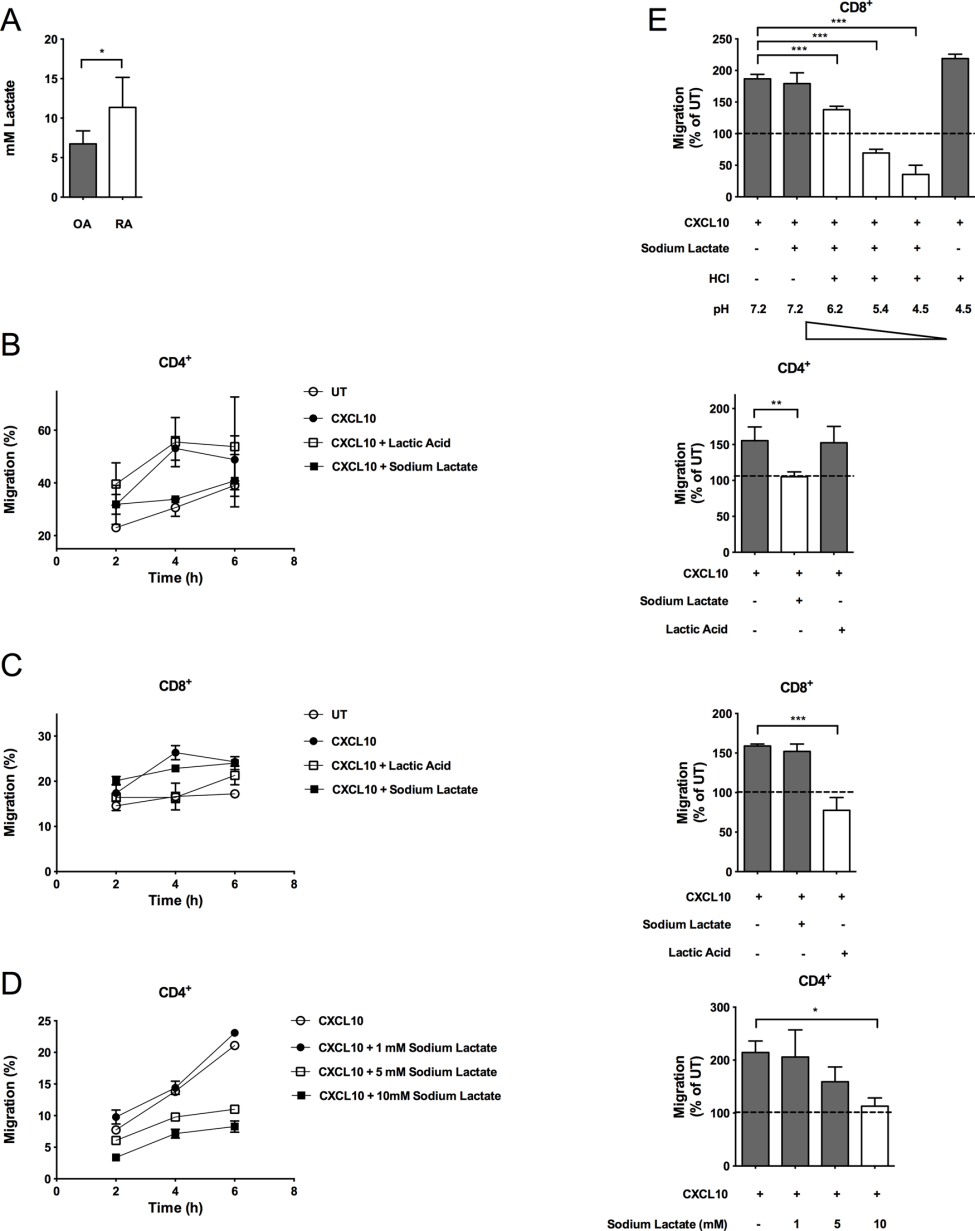
Lactate inhibits T cell motility. (A) Lactate measurements in the synovial fluid of osteoarthritis (OA) or RA patients. (B–C) In vitro chemotaxis of activated CD4^+^ (B) and CD8^+^ (C) T cells towards CXCL10 (300 ng/ml) in the presence of lactic acid (10mM) or sodium lactate (10 mM) shown as kinetic (left panel) and 4 h time point (right panel). (D) In vitro chemotaxis of activated CD4^+^ T cells towards CXCL10 (300 ng/ml) in the presence of increasing concentration of sodium lactate shown as kinetic (left panel) and 4 h time point (right panel). (E) In vitro chemotaxis (4 h time point) of activated CD8^+^ T cells towards CXCL10 (300 ng/ml) in the presence of sodium lactate (10 mM) or HCl (pH 4.5) alone, or sodium lactate in combination with increasing concentrations of HCl to obtain progressively reduced pH as indicated in figure. (A) OA, *n* = 4 and RA *n* = 8. (B right panel) *n* = 4. (C–D right panel, E) *n* = 3. (B–D left panel) Data is representative of three independent experiments; the underlying numerical data and statistical analysis for each independent experiment can be found in the supporting file, S1 Data, Fig 1B–1D. (A–E) Underlying numerical data and statistical analysis can be found in the supporting file, S1 Data, Fig 1A–1E. Values denote mean ± standard deviation (SD). * *p <*0.05; ** *p* <0.01; *** *p* <0.001.

To assess whether T cell motility is affected by lactate, we performed assays whereby chemotaxis of T cells activated for 5 d with anti-CD3 and anti-CD28 antibodies, and interleukin (IL)-2 was induced by the proinflammatory chemokine CXCL10 in the presence of 10 mM lactic acid or sodium lactate, a concentration of lactate we measured in the synovial fluid of rheumatoid arthritis (RA) patients (Fig 1A) and found in a number of inflammatory sites [14,15]. CD4^+^ T cell chemotaxis was inhibited by sodium lactate whereas that of CD8^+^ T cells was inhibited by lactic-acid but not vice versa (Fig 1B and 1C).

As T cell migration is activated by several chemokines, leading to different responses [11], we also tested the effect of lactate on chemokine (C-C motif) ligand (CCL)5-induced chemotaxis. CCL5-induced motility of CD4^+^ T cells was decreased in sodium lactate–rich but not lactic acid–rich environments (S1A Fig), suggesting a broader action of lactate in chemokine-induced signaling and downstream effects than we had anticipated in our experiments shown in Fig 1B and 1C. Motility of CD4^+^ T cells upon sodium lactate treatment decreased with increasing concentration of sodium lactate with a half maximal effective concentration (EC50) of about 10 mM sodium lactate (Fig 1D). These concentrations of sodium lactate and lactic acid did not affect cellular viability (S1B Fig).

As acidification “per se” could affect cellular motility [16], we performed additional chemotaxis assays to discriminate the effects of lactic acid on the motility of CD8^+^ T cells from the effects of pH reduction in the culture media due to the addition of 10 mM lactic acid. Lactate is present in solution either as the acid in its undissociated form (i.e., lactic acid) at low pH or as the ion salt (i.e., sodium lactate) at higher pH. We found that progressive acidification of medium containing sodium lactate (10 mM)—which increases the availability of lactic acid—reduced the motility of CD8^+^ T cells (Fig 1E). Importantly, neither the presence in the culture media of sodium lactate (10 mM) alone nor acidifying the culture media to pH 4.5 with HCl alone had any effects on the motility of CD8^+^ T cells (Fig 1E). In line with recently reported findings in macrophages [17], our data show that inhibition of CD8^+^ T cell motility by lactic acid cannot be simply ascribed to its effect on the acidification of the culture media; rather simultaneous availability of lactate and H^+^ is necessary to regulate the motility of CD8^+^ T cells. This effect did not apply to CD4^+^ T cells since their migration was not affected in lactic acid–rich medium (Fig 1B).

### The Effects of Lactate Action on Different T Cell Subsets Are Mediated by Distinct Transporters

**Fig 2:**
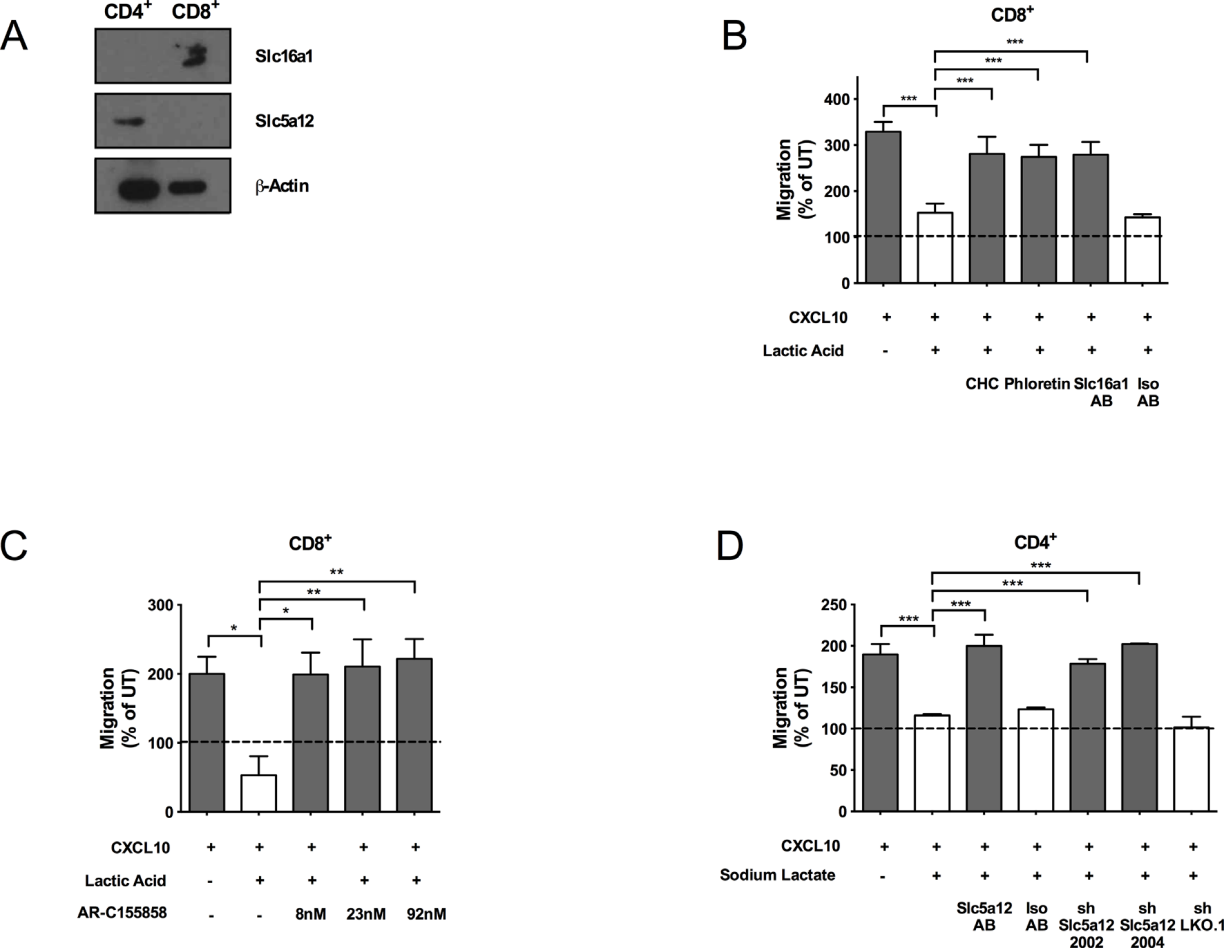
Sodium lactate and lactic acid act on CD4^+^ and CD8^+^ T cell subsets, respectively, through specific cell membrane transporters. (A) Total protein levels of the transporters Slc16a1 and Slc5a12 as assessed by western blot in activated CD4^+^ and CD8^+^ T cell subsets. (B–D) In vitro chemotaxis (4 h time point) of activated CD8^+^ T cells towards CXCL10 (300 ng/ml) in the presence of lactic acid (10 mM) alone, or in combination with α-cyano-4-hydroxycinnamate (CHC) (425 μM), phloretin (25 μM), or anti-Slc16a1 antibody (2.5 μg/ml) (B), or increasing concentrations of AR-C155858 as indicated in the figure (C), and activated CD4^+^ T cells towards CXCL10 (300 ng/ml) in the presence of sodium lactate (10 mM) alone, or in combination with an anti-Slc5a12 antibody (2.5 μg/ml) or two specific short hairpin RNAs (shRNAs) (D). An isotype control antibody has been included to control for antibody specificity (B, D), and a nonspecific shRNA has been included to control for gene knockdown specificity (D). (B–D) *n* = 3. Underlying numerical data and statistical analysis can be found in the supporting file, S1 Data, Fig 2B–2D. Values denote mean ± SD. *p < 0.05; **p < 0.01; ***p < 0.001.

We next investigated the molecular basis of the differential and mutually exclusive responsiveness of CD4^+^ and CD8^+^ T cells to sodium lactate or lactic acid, respectively. Fischer et al. described the expression by human cytotoxic lymphocytes of Slc16a1 (also known as monocarboxyl transporter [Mct]1), a lactate-H^+^ symporter which facilitates lactic acid uptake [7]. Slc5a12 is a sodium-coupled lactate transporter [18]. We found that murine CD8^+^ and CD4^+^ T cells selectively express Slc16a1 (NM_009196.4) and Slc5a12 (NM_001003915.2), respectively (Fig 2A), suggesting a specific functional role of each transporter on each T cell subset.

We subsequently sought to confirm that the differently expressed lactate transporters were functional in T cell chemotaxis inhibition. Blockade of Slc16a1 on CD8^+^ T cells with the selective inhibitors phloretin, α-cyano-4-hydroxycinnamate (CHC) [19], and AR-C155858 [20], or with a specific antibody restored chemotaxis of CD8^+^ T cells exposed to lactic acid (Fig 2B and 2C). Conversely, chemotaxis of CD4^+^ T cells in sodium lactate–rich media was recovered following selective inhibition of Slc5a12 on CD4^+^ T cells with lentiviral-delivered, specific short hairpin RNAs (shRNAs) or a specific antibody (Fig 2D and S2A Fig). As expected, the anti-Slc5a12 antibody or shRNAs targeting *Slc5a12* did not affect CD8^+^ T cell migration (S2B Fig), nor did the Slc16a1 inhibitors phloretin, CHC and AR-C155858 or the anti-Slc16a1 antibody affect migration of CD4^+^ T cells (S2C Fig).

### Sodium Lactate Limits Basal and Chemokine-Induced Aerobic Glycolysis in CD4^+^ T Cells

The lactate transporters specificity (Fig 2A) and the T cell insensitivity to lactate upon transporter inhibition (Fig 2B–2D, S2B and S2C Fig) suggest that the effects of lactate are mediated by intracellular signaling, possibly interfering with the cell metabolic machinery, and specifically with the glycolytic pathway [9], engaged downstream of chemokine receptor triggering.

**Fig 3:**
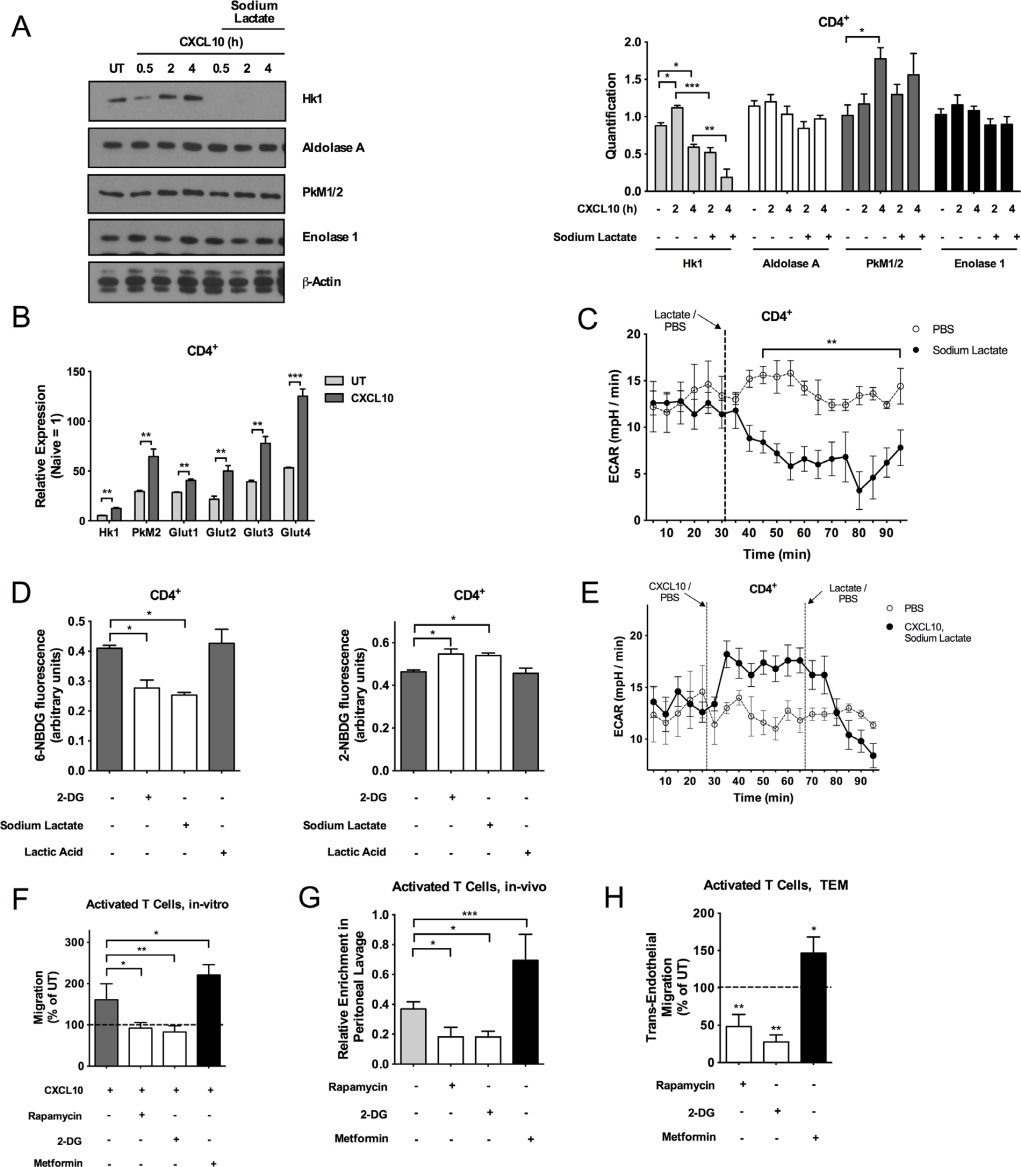
Basal and chemokine-induced aerobic glycolysis is required for CD4^+^ T cell migration. (A) Western blots with antibodies against Hk1, aldolase A, PkM1/2, enolase 1, and β-actin in activated CD4^+^ T cells treated with CXCL10 (1,000 ng/ml) alone or in combination with sodium lactate (10 mM), or left untreated. Densitometric quantification of western blots denotes mean ± SD, *n* = 3 (with biological replicates run in duplicate). *p < 0.05; ***p < 0.001. (B) Relative mRNA expression levels of *Hk1*, *PkM2*, and glucose transporters (*Glut1*, *Glut2*, *Glut3*, *Glut4*) in activated CD4^+^ T cells 6 h post-treatment with CXCL10 (1,000 ng/ml) as assessed by quantitative reverse transcription polymerase chain reaction (qRT-PCR). mRNA levels in naive CD4^+^ T cells were set to 1. (C) Extracellular acidification rate (ECAR) trace of glycolytic activity expressed as mpH/min in activated CD4^+^ T cells treated with sodium lactate (10 mM) or phosphate buffered saline (PBS). Vertical lines represent addition times of sodium lactate or PBS, respectively. (D) Measurements of glucose uptake and flux in activated CD4^+^ T cells pretreated with 2-deoxyglucose (2-DG) (1 mM), sodium-lactate (10 mM) or lactic acid (10 mM) and then incubated with the fluorescent probes 6-(N-(7-Nitrobenz-2-oxa-1,3-diazol-4-yl)Amino)-2-Deoxyglucose (6-NBDG) or 2-(N-(7-Nitrobenz-2-oxa-1,3-diazol-4-yl)Amino)-2-Deoxyglucose (2-NBDG). (E) ECAR trace of glycolytic activity in activated CD4^+^ T cells treated with CXCL10 (1,000 ng/ml) and sodium lactate (10 mM). Vertical lines represent the addition times of CXCL10, sodium lactate and PBS. (F) In vitro chemotaxis (4 h time point) towards CXCL10 (300 ng/ml) of activated CD4^+^ T cells pretreated with Rapamycin (200 nM), 2-DG (1 mM) or Metformin (2 mM). (G) Relative enrichment of IV-injected activated CD4^+^ T cells pretreated with Rapamycin (200 nM), 2-DG (1 mM) or Metformin (2 mM) and subsequently labelled with 7-Hydroxy-9H-(1,3-Dichloro-9,9-Dimethylacridin-2-One (DDAO) cell fluorescent dye in the peritoneal lavage of syngeneic recipient C57BL/6 mice i.p. injected with CXCL10 (120 ng/mouse). (H) Spontaneous transendothelial migration (6 h time point) of activated CD4^+^ T cells in the presence of rapamycin (200nM), 2-DG (1 mM) or metformin (2 mM). (B–C, E) Data is representative of three independent experiments; the underlying numerical data and statistical analysis for each independent experiment can be found in the supporting file, S1 Data, Fig 3B–3C, 3E. (D, F, H) *n* = 3. (G) *n* = 4. (A–H) Underlying numerical data and statistical analysis can be found in the supporting file, S1 Data, Fig 3A–3H. Values denote mean ± SD. *p < 0.05; **p < 0.01; ***p < 0.001.

We started by investigating the effect of CXCL10 treatment in the presence or absence of lactate on the induction of glycolysis in CD4^+^ and CD8^+^ T cells activated for 3 d with anti-CD3 and anti-CD28 antibodies and IL-2. Glycolytic enzymes are expressed in naive T cells and their expression is markedly increased upon T cell activation [21]; nevertheless, we found that CXCR3 engagement with CXCL10 was able to cause a further significant up-regulation of two rate-limiting enzymes in the glycolytic pathway, Hk1 (NM_00114100.1) and pyruvate kinase (Pk)M1/2 (NM_011099.3) (Fig 3A). Specifically, data quantification across independent experiments revealed significant up-regulation of Hk1 at 2 h and PkM1/2 at 4 h post-CXCL10 treatment, as compared to the levels expressed by activated CD4^+^ T cells left untreated (Fig 3A right). Although Hk1 appears to be up-regulated also at 4 h post CXCL10 treatment as compared to the untreated control in the western blot shown in Fig 3A left, statistical analysis across independent experiments showed a slight down-regulation of Hk1 at this point as compared to the untreated control (Fig 3A right). Similarly, *Hk1* and *PkM2* mRNA levels were up-regulated after 6 h of treatment with CXCL10 (Fig 3B). These data suggest the existence of multiple levels of regulation of the glycolytic pathway downstream of CXCR3 triggering, being both post-translational and transcriptional. In addition, activated CD4^+^ T cell exposure to CXCL10 led to increased gene expression of glucose transporters (Fig 3B) [3]. Remarkably, data quantification across independent experiments also revealed that activated CD4^+^ T cells treated with CXCL10 in the presence of sodium lactate had significantly reduced levels of Hk1 as compared to cells treated with CXCL10 alone (both at 2 and 4 h) or left untreated (only at 4 h; although a reduction appears also at 2 h in the western blot shown in Fig 3A left as compared to the untreated control, this down-regulation did not reach statistical significance across independent experiments [Fig 3A right]). In contrast, CD8^+^ T cells did not undergo major changes in the expression of glycolytic genes and proteins upon exposure to CXCL10 (S3A and S3B Fig).

Since glycolysis is selectively activated in CD4^+^ T cells upon CXCR3 triggering, we then tested the effects of sodium lactate on the glycolytic energy metabolism of CD4^+^ T cells under basal conditions by measuring the extracellular acidification rate (ECAR) in the cell culture media of CD4^+^ T cells in real time via the use of the Seahorse analyzer. We found that sodium lactate decreased the ECAR of CD4^+^ T cells from an average of 14 mpH/min in the untreated control to a level of less than 5 mpH/min (Fig 3C), indicating a decrease in glycolytic flux. In support of these data, we treated CD4^+^ or CD8^+^ T cells with the fluorescent analogues of glucose, 6-(N-(7-Nitrobenz-2-oxa-1,3-diazol-4-yl)Amino)-2-Deoxyglucose (6-NBDG), and 2-(N-(7-Nitrobenz-2-oxa-1,3-diazol-4-yl)Amino)-2-Deoxyglucose (2-NBDG), in the presence or absence of sodium lactate or lactic acid [22]. We found lower 6-NBDG-fluorescence and higher 2-NBDG-fluorescence in sodium lactate–treated CD4^+^ T cells as compared to lactic acid–treated or untreated CD4^+^ T cells suggesting that sodium lactate but not lactic acid selectively blocks glucose uptake and glycolytic flux in CD4^+^ T cells; the opposite was observed in CD8^+^ T cells (Fig 3D and S3C Fig). As expected, the inhibitor of glycolysis 2-deoxyglucose (2-DG) showed similar effects on 2-NBDG and 6-NBDG fluorescence in both T cell subsets (Fig 3D and S3C Fig), further indicating the specificity of the effects of sodium lactate and lactic acid in CD4^+^ and CD8^+^ T cells, respectively.

We next investigated whether sodium lactate was able to diminish glycolysis also upon CXCR3 engagement by CXCL10. Exposing CD4^+^ T cells to CXCL10 raised the ECAR value for several time points, indicating that the glycolytic flux is increased in these conditions (Fig 3E) [23]. Adding sodium lactate to the CXCL10-stimulated cells shut down glycolysis, as reflected by a drastic fall in ECAR (Fig 3E).

### Basal and Chemokine-Induced Aerobic Glycolysis Is Required for CD4^+^ T Cell Migration Both In Vitro and In Vivo

The down-regulation of glycolysis and the inhibitory effect on migration upon sodium lactate exposure—lactate being a direct and indirect inhibitor of glycolysis [9]—suggest that glycolysis is required for CD4^+^ T cell migration. To test this hypothesis, we treated activated CD4^+^ T cells with inhibitors or activators of glycolysis and assessed their chemotactic responses to CXCL10. Direct or indirect inhibition of glycolysis with the glucose analogue, 2-DG, or mTOR inhibitor, rapamycin (S3D Fig), caused a decrease in chemotaxis in vitro and in a well-established in vivo model of T cell recruitment to the peritoneum (Fig 3F and 3G, and S3E Fig) [24]. Conversely, activation of glycolysis via the use of the electron transfer chain Complex I inhibitor, metformin (S3D Fig) [25–28], increased chemotaxis towards CXCL10 both in vitro and in vivo (Fig 3F and 3G and S3E Fig). Accounting for the specificity of our glycolytic measurements, etomoxir, an inhibitor of fatty acid oxidation, had only minor effects on glycolysis (S3D Fig).

Metabolic drugs interfering with glycolysis had similar effects on T cell motility in spontaneous transendothelial migration assays (i.e., independent of any proinflammatory chemokine stimulus), implying the role of this pathway in steady-state control of T cell migration (Fig 3H). Importantly, exposure to the various metabolic drugs at the concentrations used did not affect the T cell surface molecule phenotype (S4 and S5 Figs).

The importance of aerobic glycolysis in activation and function of T cells has been shown previously [2,29,30], yet its potential impact on T cell migration is still unexplored; thus we assessed the impact of glycolysis on migration of naïve T cells, which mainly rely upon oxidative metabolism for their homeostasis [2]. Similar to what we had observed in activated T cells, inhibition of glycolysis via 2-DG and rapamycin resulted in a decrease in naïve T lymphocyte motility (S3F Fig), suggesting a general control of T cell migration via the glycolytic pathway. In contrast to activated T cells, however, exposure to metformin reduced naïve T cell migration (S3F Fig), indicating the existence of different metabolic checkpoints between naïve and activated T cells [31].

Overall, metabolic drugs did not affect T cell survival at the concentrations and in the experimental conditions we used (S5 Fig).

### Lactate Modulates Effector T Cell Functions

**Fig 4:**
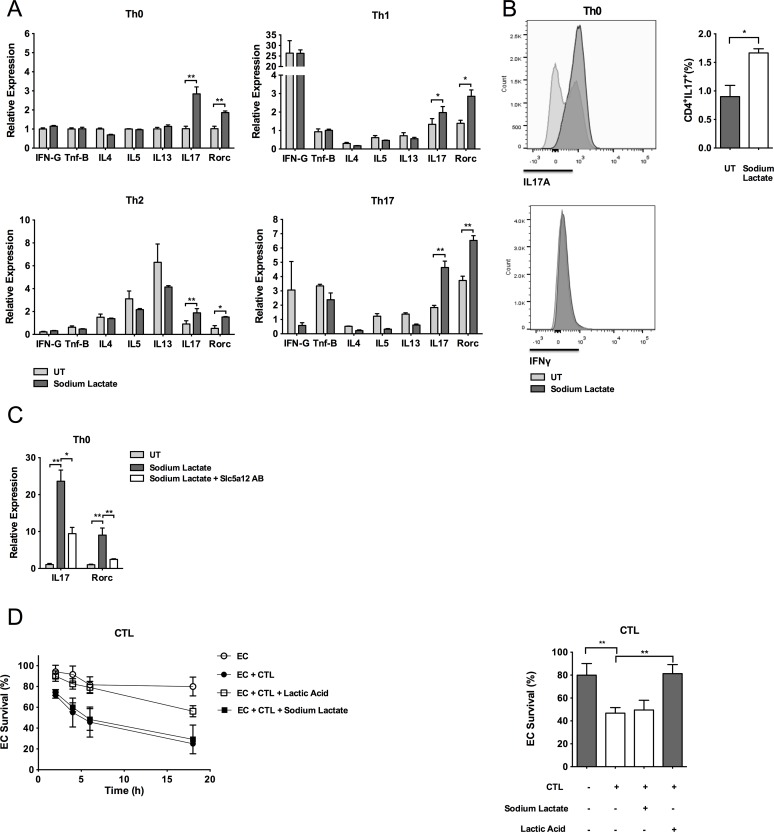
Lactate modulates effector T cell functions. (A) Relative mRNA expression levels of the cytokines interferon-gamma (*Ifn-γ*), *Tnf-β*, *Il-4*, *Il-5*, *Il-13*, and *Il-17* and of the transcription factor *Rorc* as assessed by qRT-PCR in CD4^+^ subsets Th0, Th1, Th2, and Th17 treated with sodium lactate (10 mM) or left untreated. mRNA levels of each cytokine expressed by untreated Th0 cells were set to 1. (B) Intracellular staining of IL-17A and IFN-γ in activated CD4^+^ T cells treated with sodium lactate (10 mM) or left untreated. (C) Relative mRNA expression levels of *Il-17* and *Rorc* in activated CD4^+^ T cells treated with sodium lactate alone or in combination with an anti-Slc5a12 antibody. mRNA levels of untreated T cells were set to 1. (D) Cell survival of allogeneic endothelial cells in the presence of CD8^+^ cytotoxic T cells and lactic acid (10 mM) or sodium lactate (10 mM) shown as kinetic (left panel) and 6 h time point (right panel). (A, C, D left panel) Data is representative of three independent experiments; the underlying numerical data and statistical analysis for each independent experiment can be found in the supporting file, S1 Data, Fig 4A, 4C, and 4D. (B, D right panel) *n* = 3. (A–D) Underlying numerical data and statistical analysis can be found in the supporting file, S1 Data, Fig 4A–4D. Values denote mean ± SD. *p < 0.05; **p < 0.01.

To investigate whether sodium lactate could affect CD4^+^ T cell effector functions, we induced polarization of CD4^+^ T cells towards Th1, Th2, and Th17 subsets in the appropriate cytokine “milieus” [32,33]. The expected patterns of cytokine expression by differentiated Th subsets were confirmed at the mRNA level (S6 Fig). We then tested the effect of the presence of sodium lactate on the release of cytokines by the different Th subsets in the same polarizing conditions. Gene expression analysis showed that treatment with sodium lactate caused a significant up-regulation of *Il-17* (NM_010552.3) in all the Th subsets (Fig 4A). Supporting these data, gene expression of *Rorc* (NM_011281.2), the signature transcription factor of Th17 cells, was also significantly elevated in all the Th subsets upon T cell exposure to sodium lactate (Fig 4A). Gene expression of Th1 and Th2 signature cytokines instead was unmodified upon treatment with sodium lactate (Fig 4A). Intracellular staining experiments confirmed the increased expression of IL-17 protein in CD4^+^ T cells exposed to sodium lactate as compared to cells left untreated (Fig 4B). Remarkably, preincubation with the antibody anti-Slc5a12 blocked the up-regulation of *Il-17* and *Rorc* genes induced by sodium-lactate (Fig 4C).

CTL differentiating from the CD8^+^ subset express and release cytolytic granules consisting of perforin/granzyme complexes, which promote the killing of target cells [34]. To test whether these functions were affected by lactate, we performed cytotoxicity assays with allogeneic endothelial cells. Similarly to the results obtained in migration assays (Fig 1C), lactic acid but not sodium lactate inhibited the cytolytic activity of CTL (Fig 4D).

### Inhibition of Lactate Transporters Promotes the Release of T Cells from the Inflamed Tissue

**Fig 5:**
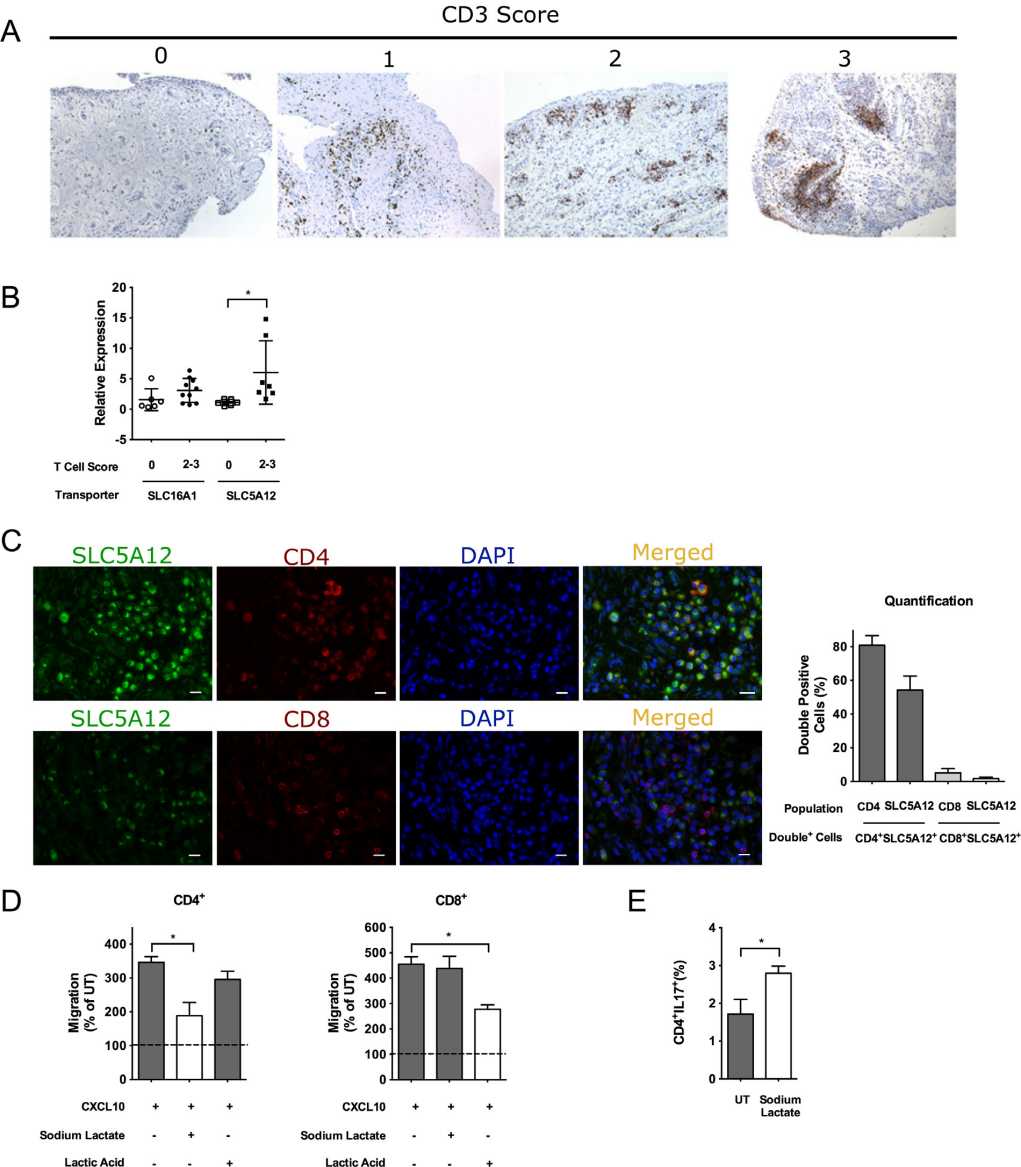
High Slc5a12 expression in RA in humans. (A) Representative images of RA synovial tissues stained for CD3 displaying progressively higher degree of T cell infiltration as quantified using a semiquantitative score from T0 (absence of infiltrating T-cells) to T3 (large number of infiltrating T cells organizing in ectopic follicles) as shown in [35]. (B) Relative mRNA expression levels of *Slc16a1* and *Slc5a12* in the synovial fluid isolated from the joints of RA patients. Samples are grouped based on their T cell score as described in A. Values denote mean ± SD, (T0) *n* = 6 and (T2–3) *n* = 7. *p < 0.05. (C) Double immunofluorescence staining for Slc5a12 and CD4 or CD8 in the synovial tissue of RA patients. Slc5a12 (green) is highly expressed within the RA synovia in the presence of a high degree of CD4^+^ (red) T cell infiltration. Merging (yellow) of the green and red channels demonstrates that Slc5a12 is selectively expressed by CD4^+^ but not CD8^+^ infiltrating T cells. Quantification of the % double positive cells is provided upon counting positive cells (single and double positive for each marker) in at least 6 images per condition. Columns represent % of double positive CD4^+^ Slc5a12^+^ population within the CD4^+^ or Slc5a12^+^ cells and % of double positive CD8^+^ Slc5a12^+^ population within the CD8^+^ or Slc5a12^+^ cells. Scale bars: 50 μm. (D) In vitro chemotaxis (4 h time point) of activated human CD4^+^ and CD8^+^ T cells towards CXCL10 (300 ng/ml) in the presence of lactic acid (10 mM) or sodium lactate (10 mM). (E) Intracellular staining of IL-17A in activated human CD4^+^ T cells treated with sodium lactate (10 mM) or left untreated. (D) *n* = 3. (E) *n* = 4. (B–E) Underlying numerical data and statistical analysis can be found in the supporting file, S1 Data, Fig 5B–5E. Values denote mean ± SD. *p < 0.05.

We have shown that in humans the synovial fluid of RA contains elevated levels of lactate compared with noninflammatory types of arthritis (e.g., osteoarthritis [OA], Fig 1A). The rheumatoid synovial environment is paradigmatic of all the lactate-induced changes in T cells, including entrapment, IL-17 secretion and loss of antigen responsiveness [35]. We therefore investigated the expression and cellular localization of Slc5a12 and Slc16a1 within the synovial tissue of 16 patients suffering from RA (S1 Table, demographical data). We first stratified RA patients for the amount of CD3^+^ infiltrating T cells using a semiquantitative score (Fig 5A) as we previously described [35]. Next, we performed gene expression analysis and found that *Slc5a12* mRNA expression significantly increased in correlation with the T cell score of the samples tested (Fig 5B). Albeit not significant, a trend towards increased *Slc16a1* expression could also be observed in CD8^+^ T cells (Fig 5B). In order to confirm our in vitro data that Slc5a12 is expressed on CD4^+^ but not CD8^+^ T cells (Fig 2A), we performed double immunofluorescence for Slc5a12 and either CD4 or CD8. As shown in Fig 5C, within the RA synovial tissue Slc5a12 is abundantly and selectively expressed by CD4^+^ but not CD8^+^ T cells. Enhanced expression of the Slc5a12 transporter by CD4^+^ T cells in the RA synovia opens the possibility that this transporter might be mediating the migratory and functional changes that we have previously described and that correlate with key features of T cell infiltrates in RA. In support to this, we found that CD4^+^ and CD8^+^ T cells isolated from the peripheral blood of human healthy donors responded in vitro to sodium lactate and lactic acid similarly to their murine counterparts in terms of migration (i.e., activated human CD4^+^ and CD8^+^ T cells; Fig 5D) and expression of IL-17 protein (i.e., activated human CD4^+^ T cells upon exposure to sodium lactate as compared to cells left untreated; Fig 5E).

**Fig 6:**
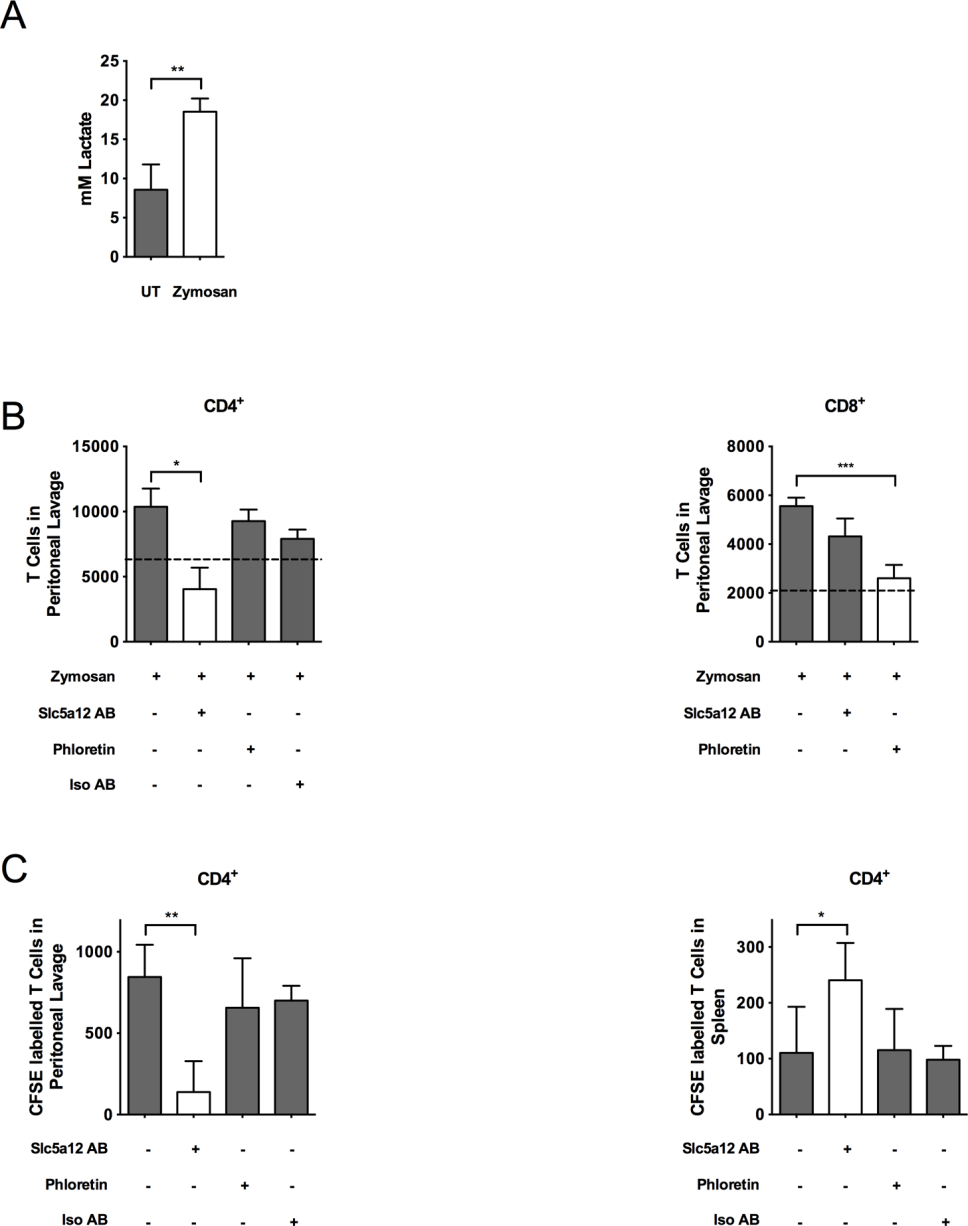
Inhibition of lactate transporters promotes the release of T-cells from the inflamed site in zymosan-induced peritonitis. (A) Lactate levels in the peritoneum of zymosan-treated mice. (B) Number of CD4^+^ and CD8^+^ T cells, respectively, in the peritoneal lavage of C57BL/6 mice injected i.p. with zymosan (1 mg/mouse) to induce peritonitis, and 5 d later, i.p. treated with phloretin (50 μM), an anti-Slc5a12 antibody (5 μg/ml) or an isotype control antibody. (C) Number of carboxyfluorescein succinimidyl ester (CFSE)-labeled activated CD4^+^ T cells in the peritoneal lavage (left panel) or spleen (right panel), respectively, of C57BL/6 mice injected i.p. with zymosan (1 mg/mouse), then i.p. treated with phloretin (50 μM), an anti-Slc5a12 specific antibody (5μg/ml) or an isotype control antibody. (A–C) *n* = 3 or more. Underlying numerical data and statistical analysis can be found in the supporting file, S1 Data, Fig 6A–6C. Values denote mean ± SD. *p < 0.05; **p < 0.01; ***p < 0.001.

Prompted by these results, we sought to assess whether lactate promotes the retention of T cells into inflammatory sites in vivo and whether inhibitors of the lactate transporters favor the release of T cells from the inflamed site. We used a well-established mouse model of zymosan-induced peritonitis, in which T cells are recruited to the inflamed site 5 d after zymosan injection [36]. C57BL/6 mice were injected in the peritoneal cavity with zymosan (1 mg/mouse) on day 0 or left untreated. On day 5, phloretin, an anti-Slc5a12 antibody or an isotype control antibody were injected into the peritoneal cavity. 24 h later, mice were killed and the peritoneal lavage was harvested. Lactate levels and CD4^+^ and CD8^+^ T cells were increased significantly in the peritoneum of recipient animals (Fig 6A and 6B). Intraperitoneal injection of anti-Slc5a12 antibody caused a significant reduction of CD4^+^ T cells in the peritoneum in comparison to an isotype control antibody, while having no effect on CD8^+^ T cells (Fig 6B, S7A, and S7B Fig). In contrast, phloretin promoted a significant decrease of CD8^+^ T cells in the peritoneum but did not show any effect on CD4^+^ T cells (Fig 6B, S7A, and S7B Fig).

To establish that the decrease in T cell localization to the peritoneal cavity was at least in part due to their increased egression from this site, we performed adoptive transfer experiments whereby carboxyfluorescein succinimidyl ester (CFSE)-labelled CD4^+^ T cells were coinjected with anti-Slc5a12 antibody, phloretin, or an isotype control antibody in the peritoneal cavity of mice which had received zymosan (1 mg/mouse) 5 d before, to create an environment rich of lactate (Fig 6A). 24 h after the intraperitoneal injection of CFSE-labelled CD4^+^ T cells, mice were killed, and peritoneal lavage and spleen were harvested. Injection with anti-Slc5a12 antibody but not phloretin or the isotype control antibody in the peritoneal cavity caused a reduction of adoptively transferred T cells in the peritoneum and their accumulation in the spleen (Fig 6C and S7C Fig).

## Discussion

CIDs are a major public health concern in the western world. The nonresolving nature of inflammation in CID, associated with excessive and inappropriate activation of the immune system, is pivotal to the disease process and represents a treasure trove for drug discovery via the identification of novel processes and molecules that could be targeted for the design of new therapeutics.

In this study, we have identified a key role for the glycolytic pathway and its metabolite, lactate, in the regulation of T cell migration and functions in inflammation. Lactate can be imported into the cell through specific transporters and acts as a potent inhibitor of two of the rate-limiting enzymes in the glycolytic pathway, Hk (catalyzing the first reaction) and Pfk (the key enzyme in setting the pace of glycolysis, which catalyzes the third reaction), but does not affect Pk (the third rate-limiting enzyme in glycolysis, which catalyzes the tenth and last reaction) [9]. Consistently, our data show lactate-induced down-regulation of Hk1 but not PkM1/2 protein levels. Unlike Hk1 and PkM1/2, we did not find any significant regulation of Pfk upon CXCL10 stimulus. Nevertheless, our data shows that lactate down-regulates Hk1, which is sufficient to reduce the glycolytic flux and in turn causes reduced T cell migration. In recent years, aerobic glycolysis has been associated with cell division in a number of physiological (e.g., immunity, stem cell compartment) [31,37,38] and pathological (e.g., cancers) processes [39,40]. Very recently, however, a metabolic switch to aerobic glycolysis has been linked to more specialized–independent of proliferation–cell functions, such as interferon-gamma (IFN-γ) production in T cells [29] and vessel sprouting in endothelial cells [41]. In the latter study, glycolysis has been directly linked to cytoskeleton reorganization and migration of endothelial cells during neoangiogenesis via the glycolytic activator 6-phosphofructo-2-kinase/fructose-2,6-biphosphatase 3 (PFKFB3). Up to date, a direct link between T cell migration and intracellular metabolic pathways had not been established [3,4]. Our study provides direct evidence that intracellular metabolism, specifically glycolysis, is required for chemokine-induced T cell migration. Further studies will be required to identify specific factors and pathways to causally link the induction of aerobic glycolysis that happens during T cell migration in response to the chemokine stimulus to actin and cytoskeleton reorganization as well as its physiological impact.

The effect of lactate on T cells is exerted via transporters, which are differentially expressed by CD4^+^ and CD8^+^ T cells. This is a previously unknown feature that differentiates these two subsets. As T cells are likely to be exposed to lactate as a consequence of the high proliferation rates in the thymus, it is possible that the expression of either transporter is determined and functional during development and might be regulated by other developmental signals, such as TCR engagement. The physiological significance of distinct lactate transporter expression by CD4^+^- and CD8^+^-activated T cells might dictate their functional and migratory responses depending on the nature of the inflammatory exudate (i.e., more lactic acid versus sodium lactate) and underlay the differential distribution of these T cell subsets in the inflamed tissue, as it has been described in tumours and CIDs [42–44]. Clarifying the molecular mechanisms and physiological significance of this differential expression will require further investigations.

**Fig 7:**
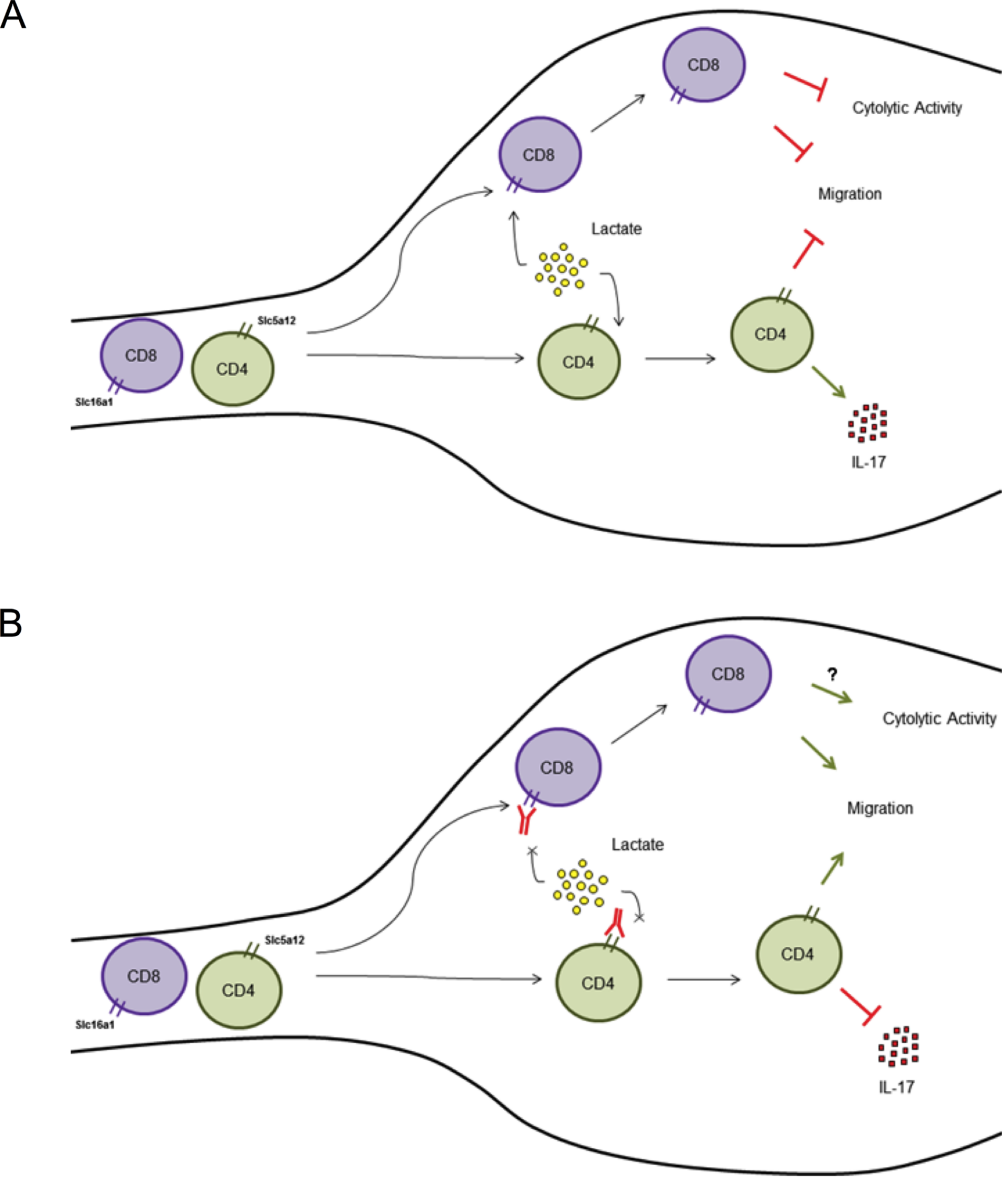
Schematic of the proposed mechanism of lactate effects on T cells in the inflammatory site. (**A**) The motility of CD4^+^ and CD8^+^ T cells is blocked once they get exposed to elevated levels of lactate in the inflammatory site. Lactic acid also causes loss of cytolytic activity by CD8^+^ T cells, and sodium lactate promotes the production of IL-17 by CD4^+^ T cells. (**B**) Pharmacologic targeting of lactate transporters re-establish T cell migration away from the inflammatory site and block the production of high amounts of IL-17.

The effect of lactate on T cells recapitulates key features of T lymphocytes found in chronic inflammatory infiltrates, including their entrapment and production of high levels of IL-17 and loss of cytolytic activity by CD4^+^ and CD8^+^ T cells, respectively (Fig 7A) [2,12]. We show that sodium lactate induces all CD4^+^ Th subsets to increase their production of the proinflammatory cytokine IL-17, which is prevalent in the inflamed tissue and is known to drive forward the chronic inflammatory process in many CIDs [2,12]. Previous reports have shown that HIF-induced glycolysis is necessary to promote the differentiation of naïve CD4^+^ T cells toward the Th17 subset [45]. We have analyzed memory T cells already committed to a specific subset, similar to T cells in the inflammatory site. After in vitro incubation with sodium lactate, these cells acquire a mixed phenotype whereby they continue to express their own signature cytokines but at the same time increase the expression of Rorγt and IL-17. In this context, it has recently been suggested that increased salt (sodium chloride, NaCl) concentrations found locally under physiological conditions in vivo markedly boost the induction of murine and human Th17 cells in autoimmune conditions [46]. Our results expand this observation to a broader range of salts, including sodium lactate. In keeping with the in vitro data, we also show that in the chronically inflamed synovial tissue of patients with RA, a disease associated with high levels of IL-17^+^/CD4^+^ T cells in the joints [47], infiltrating CD4^+^, but not CD8^+^, T cells almost invariably displayed high expression of Slc5a12. This suggests that the expression of Slc5a12 on the CD4^+^ subset may be involved in the entrapment of CD4^+^ T cells within the chronically inflamed “milieu” of the RA synovia (Fig 7A). In this context, we show that targeting the lactate transporters Slc16a1 and Slc5a12 re-establish T cell migration away from the inflammatory site and block the production of high amounts of IL-17, with potential therapeutic implications in the management of CIDs (Fig 7B). The mechanisms for lactate-mediated induction of IL-17 production are at present unclear; further studies will be required to assess whether such control occurs at metabolic level. These findings also suggest the idea that elevated lactate concentrations within an inflamed site might sustain persistent inflammation by reducing T lymphocyte cytolytic killing activity (Fig 7A) [33].

In summary, our observations suggest that lactate-induced signaling in the inflammatory “milieu” may promote many of the pathogenic features and persistence of the T cell infiltrates. Importantly, we show that pharmacologic targeting of lactate transporters in T cells might provide a novel and viable approach to resolve chronic T cell–mediated inflammation in CIDs.

## Materials and Methods

### Ethical Statement

Human blood was obtained from healthy donors according to ethics approval from Queen Mary Ethics Research Committee (QMERC2014/61). RA synovial tissue was collected after informed consent according to ethics approval from Barts and the London NHS Trust (LREC 07/Q0605/29). All in vivo experiments were conducted under the UK Home Office regulation (PPL 70/7443).

### Chemicals and Reagents

Chemicals and reagents were purchased from Sigma-Aldrich & Co (UK), unless otherwise specified.

### T Cell Isolation, In Vitro Activation and Subset Enrichment

Murine T cells were isolated from lymph nodes of C57BL/6 mice and activated for 3 to 5 d with plate bound anti-CD3 (1 μg/ml) and anti-CD28 (5 μg/ml) antibodies (BioLegend), and IL-2 (10 ng/ml; PeproTech). Murine CD4^+^ and CD8^+^ T cell subsets were isolated with commercially available isolation kits (negative selection; Easysep, Invitrogen) according to manufacturer’s instructions either prior or post activation according to experimental settings. Human PBMCs were isolated from blood using a single density centrifugation (Histopaque 1077, Sigma-Aldrich). CD4^+^ or CD8^+^ T cells were then isolated from the PBMCs (negative isolation) using Dynabeads^®^ magnetic separation (Invitrogen), according to manufacturer’s instructions. Untouched human CD4^+^ and CD8^+^ T cells were stimulated for 5 d with 0.5 μg/ml of soluble anti-CD3 (clone Hit3a) and 0.5 μg/ml of anti-CD28 (clone CD28.2) (Biolegend), together with 10 ng/ml IL-2 (Peprotech).

### Chemotaxis Assays

Chemotaxis assays were performed in 5 μm transwell inlays (Corning). In some experiments, T cells were pretreated overnight with a number of drugs purchased from Calbiochem: Rapamycin (200 nM), 2-DG (1 mM), Metformin (2 mM). In most experiments, 1 h before the assay cells were incubated with lactic acid (10 mM) or sodium lactate (10 mM), either alone or in combination with Phloretin (25 μM, corresponding to its Ki [inhibitor constant] for Slc16a1 (MCT1); phloretin can also target MCT4 with a Ki of 41 μM, thereby excluding issues of specificity at the concentration we used [19]), CHC (425μM, corresponding to its Ki for Slc16a1; CHC can also target MCT4 with a Ki of 991 μM, thereby excluding issues of specificity at the concentration we used [19]), AR-C155858 (8, 23, or 92 nM [Tocris Bioscience], covering its IC50 of around 20 nM for Slc16a1 [20]; AR-C155858 binds to MCT2 with similar affinity as to Slc16a1, but MCT2 is not expressed in CD8^+^ T cells [7]), Slc5a12 specific antibody (2.5 μg/ml; Abcam) or Slc16a1 specific antibody (2.5 μg/ml; Abcam). 3 x 10^5^ lymphocytes suspended in migration medium (RPMI 2% FCS) were seeded in the upper transwell chamber; chemokines (PeproTech) were added to the lower chamber: CXCL10 (300 ng/ml), CCL5 (50 ng/ml), CCL19/21 (200 ng/ml of each chemokine). Migrated T cells were counted with a hemocytometer 2, 4, and 6 h after seeding, then percent of migrated cells were calculated. In some assays, microvascular endothelial cells (isolated from the lungs of C57BL/6 mice) were seeded (3 x 10^4^/well) on 3 μm transwell inlays. On the following day, T cells (3 x 10^5^/well) suspended in migration medium were added to the top chamber of the transwell and incubated at 37°C with 5% CO_2_ to allow migration through the endothelial monolayers. Migrated T cells were counted with a hemocytometer 6 h after seeding, then percent of migrated cells was calculated.

### RNA Isolation and Reverse Transcription

RNA was isolated from 10^6^ cells or 10 mg RA synovial tissue using commercially available kits (Qiagen) or Trizol (Life) according to the manufacturer’s instructions and assessed for quality and quantity using absorption measurements. Reverse transcription to cDNA was performed according to the manufacturer’s instruction (Applied Biosystems).

### qRT-PCR

Gene expression analysis was done using SYBR Green Supermix (Biorad) in CFX connect light cycler (Biorad), according to the manufacturer’s instructions. Gene-relative expression was calculated using the ΔΔct method [48] and normalized to a reference control (Rplp0). Primers for qRT-PCR were designed with the assistance of online tools (Primer 3Plus) using at least one exon junction binding site per primer pair where possible. A complete list of primers used is available in the supporting information (S2 Table).

### Western Blot

Protein lysates were prepared from activated T cells in RIPA buffer. Proteins were separated with SDS-PAGE and transferred to a Nylon membrane (GE Healthcare). Membranes were blocked for 2 h at room temperature in 5% Milk/TBS-T, incubated overnight at 4°C with primary antibodies (1:1,000) and subsequently with HRP-conjugated secondary antibody (Amersham Bioscience) (1:5,000). Antibodies against Hk1, Pk M1/2, Aldolase A, Enolase 1, and β-actin were purchased from Cell Signaling; antibodies against Slc16a1 and Slc5a12 were purchased from Abcam. Density of bands of glycolytic enzymes was calculated relative to β-actin via the use of Image J software.

### Lentivirus Preparation

Bacterial glycerol stocks containing shRNA plasmid clones targeting Slc5a12 were purchased from Sigma and grown in Luria Bertani broth. Plasmids were isolated using Plasmid Maxi kit (Qiagen). HEK293T-cells were grown in 10 x 10 cm cell culture dishes to 70% confluence and transfected with plasmids using the calcium phosphate method. The supernatant was harvested 48 and 72 h after transfection and 100-fold concentrated in an ultracentrifuge. Aliquots were stored at −80°C.

### Lentiviral Transduction and sh-RNA-Mediated Gene Silencing

Primary CD4^+^ T cells were isolated from C57BL/6 murine lymph nodes and activated with plate bound anti-CD3 and anti-CD28, and IL-2 for 3 d. On day 3, medium was changed and cells were incubated with 25 μl virus/10^6^ cells in the presence of polybrene (8 μg/ml). Virus was removed 24 h later; T cells were washed twice with PBS and incubated for 24 h in complete RPMI culture media.

### Measurement of Lactate, Glycolysis, Glucose Uptake/Flux and Cell Death

Lactate concentration was measured in the synovial fluid or peritoneal lavage using the Lactate assay Kit (Biovision), according to the manufacturer’s instructions. Glycolytic metabolism was measured with a Seahorse XF24 Extracellular Flux Analyzer. Briefly, T cells were grown in high glucose RPMI-1640 supplemented with 10% FCS. One hour before the experiment, 5 x 10^5^ T cells were seeded in a 24-well microplate in XF Assay Modified DMEM, and CXCL10, sodium lactate, metabolic drugs, or PBS were injected during measurement. For glucose uptake and flux assays, 6-NBDG and 2-NBDG (Life), two fluorescent analogues of glucose that are taken up by cells via glucose transporters, were used. Their fate differs upon cellular uptake. 6-NBDG cannot be phosphorylated by hexokinase, the enzyme that initiates glycolysis, and accumulates in the cytoplasm in its fluorescent form. 2-NBDG instead is phosphorylated by hexokinase and transformed in nonfluorescent intermediates of the glycolytic pathway with kinetics dependent on the pace of the glycolytic flux in the cell [49]. Therefore, parallel measurements of 6-NBDG and 2-NBDG fluorescence provide a read out of glucose uptake and glycolytic flux in cells. T cells were pre-treated with 2-DG, sodium-lactate or lactic-acid for 1 hour and then incubated with the fluorescent probes 2-NBDG for 30 minutes or 6-NBDG for 10 minutes. At the end of these treatments cells were harvested, washed twice with PBS and fluorescence read according to the manufacturer’s instructions. T-cell viability upon lactate or metabolic drug treatment was assessed by trypan blue exclusion assay.

### CTL Differentiation and Activity Assay

Isolated CD8^+^ T cells (BALB/c) were incubated with CD3-depleted and mytomycin-C-eradicated allogeneic splenocytes (C57BL/6). Differentiated CTLs were enriched with Ficoll and CD3 enrichment kits and cocultured with endothelial cells (C57BL/6) in the presence or absence of 10 mM lactic acid or sodium lactate. Dead cells were counted using trypan blue exclusion assay 2, 4, 6, and 18 h after the start of the assay.

### Th Subset Differentiation

T cells were isolated from murine lymph nodes and enriched for CD3^+^ and subsequently CD4^+^ subsets. 10^6^ cells were plated/well and differentiated towards Th0, Th1, Th2, and Th17 phenotypes. Conditions were: Th0 (10 ng/ml IL-2); Th1 (10 ng/ml IL-2; 3.4 ng/ml IL-12; 2 μg/ml Anti-IL-4); Th2 (10 ng/ml IL-2; 10 ng/ml IL-4; 2 μg/ml Anti-IFN-γ); Th17 (10 ng/ml IL-6; 2 μg/ml Anti-IL-4; 2 μg/ml Anti-IFN-γ, 5 ng/ml TGF-β). All antibodies and cytokines were purchased from PeproTech.

### Intracellular Protein Staining

Murine T cells were incubated in permeabilization/fixation buffer (ebioscience) overnight at 4°C. Samples were washed in permeabilization buffer (ebioscience) and stained for the cytokines IFN-γ and IL-17A, using fluorescently conjugated primary antibodies (1:200, ebiosciences) at 4°C for 30 min. Human-isolated CD4^+^ T cells were treated as described above. In the final 4 h of culture, cells were treated with 50 ng/ml PMA and 500 ng/ml ionmycin and 1:1,000 Brefeldin A (Sigma-Aldrich), followed by surface staining for CD4 (clone RPA-T4, Biolegend). Cells were fixed and permeabilized as described above, followed by staining for IL-17A (clone BL168, Biolegend) for 30 min at room temperature. All intracellular staining was assessed by flow cytometry using a LSR Fortessa (BD Biosciences) and FlowJo version 7.6.5 software.

### Human RA Synovial Tissue Collection and Immunohistology/ Immunofluorescence

RA synovial tissue was collected from a total of 16 RA patients undergoing total joint replacement or ultrasound-guided synovial biopsies as previously described [50]. A summary of the demographical and clinical characteristics of the RA patients is reported in S1 Table. For total T cell scoring, paraffin sections were stained for CD3, and a semiquantitative score was applied as previously described [35]. For Slc5a12 single and double (with CD4 or CD8) immunofluorescence, after antigen retrieval (S2367, Dako) and block of nonspecific binding, slides were incubated with primary antibodies either overnight at 4°C (CD4 and CD8, 1:50, Dako) or 1 h at RT (Slc5a12, 1:50, Novus Biologicals) followed by fluorochrome-conjugated secondary antibodies (Invitrogen, Eugene, Oregon, USA). All sections were visualised using a Zeiss fluorescence microscope. Quantification was performed by calculating the percent of double positive CD4^+^ Slc5a12^+^ population within the CD4^+^ or Slc5a12^+^ cells and the percent of double positive CD8^+^ Slc5a12^+^ population within the CD8^+^ or Slc5a12^+^ cells. The number of positive cells (single and double positive for each marker) was counted in six images per condition.

### In Vivo Peritoneal Recruitment Model

Activated T cells (5 x 10^6^/mouse) were pretreated overnight with Rapamycin (200 nM), 2-Deoxyglucose (1 mM), or Metformin (2 mM), then labelled with the fluorescent cell dye DDAO (Invitrogen) and injected intravenously into syngeneic female C57BL/6 mice that had 3 h prior received an intraperitoneal injection of CXCL10 (1,200 ng/mouse). 24 h after injection, mice were killed and spleen and peritoneal lavage were harvested. T cells were stained for surface markers (CD4 and CD8, ebiosciences) and analysed by FACS. Cells were first gated on CD4 and subsequently analysed for DDAO positivity. This method was used in Fig 3G and S3E Fig.

### In Vivo Zymosan-Induced Peritonitis

C57BL/6 mice were injected in the peritoneal cavity with zymosan (1 mg per mouse) on day 0 or left untreated. On day 5, Phloretin (50 μM), anti-Slc5a12 antibody (5 μg/ml) or anti-rabbit IgG isotype control antibody (5 μg/ml; Invitrogen) were injected into the peritoneal cavity. 24 h later, mice were killed and the peritoneal lavage was harvested. T cells were stained for surface markers (CD4 and CD8; ebiosciences) and analyzed by FACS. This method was used in Fig 6B and S7A and S7B Fig. Alternatively C57BL/6 mice were injected in the peritoneal cavity with zymosan (1 mg per mouse) on day 0. On day 5, activated CD4^+^ T cells (5 x 10^6^/mouse) labelled with the fluorescent cell dye CFSE (3.3μM; Invitrogen) were coinjected with anti-Slc5a12 antibody (5 μg/ml), phloretin (50 μM), or an isotype control antibody (5 μg/ml) in the peritoneal cavity. 24 h later, mice were killed and the peritoneal lavage and the spleen were harvested. T cells were stained for surface markers (CD4 and CD8; ebiosciences) and analyzed by FACS. Cells were first gated on CD4 and subsequently analysed for CFSE positivity. This method was used in Fig 6C and S7C Fig.

### FACS

Isolated T cells were stained for surface markers; CD3, CD4, CD8, CD25, CXCR3, CCR7, CD62L, and LFA-1 with fluorescently conjugated primary antibodies (1:200, ebiosciences) at 4°C for 30 min and assessed by flow cytometry using a LSR Fortessa (BD Biosciences) and FlowJo version 7.6.5 software.

### Statistical Analysis

Data are expressed as mean ± standard error of the mean (SEM). Two-tailed Student’s *t* test was used to compare two groups with parametric data distribution. For multiple comparison analysis, 1-, 2- or 3-way ANOVA was used. In all cases, a *p*-value of less than 5% was considered to be significant.

## Supporting Information

S1 Data Excel spreadsheet containing, in separate sheets, the underlying numerical data and statistical analysis for Figs 1A–1E, 2B–2D, 3A–3H, 4A–4D, 5B–5E, and 6A–6C. (XLSX)

S2 Data Excel spreadsheet containing, in separate sheets, the underlying numerical data and statistical analysis for S1A–S1B, S2A–S2C, S3A–S3D, S3F, and S6 Figs. (XLSX)

S1 Fig Lactate inhibits T cell migration upon CCL5 stimulus and does not affect cellular viability. (A) In vitro chemotaxis of activated CD4^+^ T cells towards CCL5 (50 ng/ml) in the presence of lactic acid (10 mM) or sodium lactate (10 mM), shown as kinetic (left panel) and 4 h time point (right panel). (B) Total cell number of viable CD4^+^ T cells treated with CXCL10 in the presence of lactic acid (10 mM) or sodium lactate (10 mM). (A left panel) Data is representative of three independent experiments; the underlying numerical data and statistical analysis for each independent experiment can be found in the supporting file, S2 Data, S1A Fig (A right panel, B) *n* = 3. (A–B) Underlying numerical data and statistical analysis can be found in the supporting file, S2 Data, S1A–S1B Fig Values denote mean ± SD. ***p < 0.001.
(TIFF)

S2 Fig Inhibition of T cell migration via blockade of lactate transporters is sub-type specific. (A) Western blots and qRT-PCR with *Slc5a12*-specific primers and RNAs from activated CD4^+^ T cells expressing the shRNAs shown. (B, C) In vitro chemotaxis (4 h time point) of activated CD8^+^ T cells towards CXCL10 in the presence of lactic acid (10 mM) alone or in combination with an anti-Slc5a12 antibody (2.5 μg/ml) or an isotype control antibody (B left panel) and two specific shRNAs for *Slc5a12* or a nonspecific shRNA (B right panel), and activated CD4^+^ T cells towards CXCL10 in the presence of sodium lactate alone or in combination with CHC (425 μM) or phloretin (25 μM) (C left panel) and an anti-Slc16a1 (2.5 μg/ml) or an isotype control antibody, or AR-C155858 (8 nM) (C right panel). (A) Data is representative of three independent experiments; the underlying numerical data and statistical analysis for each independent experiment can be found in the supporting file, S2 Data, S2A Fig (B–C) *n* = 3. (A–C) Underlying numerical data and statistical analysis can be found in the supporting file, S2 Data, S2A–S2C Fig Values denote mean ± SD.*p < 0.05; ** *p* < 0.01; ***p < 0.001.
(TIFF)

S3 Fig Glycolysis and chemotaxis in naïve and activated CD4^+^ and CD8^+^ T cells. (A) Western blots with antibodies against Hk1, aldolase A, PkM1/2, enolase 1, and β-actin in activated CD8^+^ T cells treated with CXCL10 (1,000 ng/ml) or left untreated. Densitometric quantification of western blots denotes mean ± SD, *n* = 3 (with biological replicates run in duplicate). (B) Relative mRNA expression levels of *Hk1*, *PkM2*, and glucose transporters (*Glut1*, *Glut2*, *Glut3*, and *Glut4*) in activated CD8^+^ T cells 6 h post-treatment with CXCL10 (1,000 ng/ml) as assessed by qRT-PCR. mRNA levels in naive T cells were set to 1. (C) Measurements of glucose uptake and flux in activated CD8^+^ T cells pretreated with 2-DG, sodium lactate or lactic acid and then incubated with the fluorescent probes 6-NBDG or 2-NBDG. (D) ECAR trace of glycolytic activity expressed as mpH/min in activated CD4^+^ T cells treated with 2-DG (1 mM), Rapamycin (200 nM), Metformin (2 mM) or Etomoxir (100 μM). (E) Representative FACS dot plots of DDAO-labelled donor CD4^+^ T cells collected from the peritoneal lavage and spleen of recipient mice, which correspond to the relative enrichment in peritoneal lavage shown in Fig 3G. (F) In vitro chemotaxis (4 h time point) towards the chemokines CCL19/21 (200 ng/ml of each chemokine) of naïve T cells pretreated with Rapamycin (200 nM), 2-DG (1 mM) or Metformin (2 mM). (B, D) Data is representative of three (B) and two (D) independent experiments; the underlying numerical data and statistical analysis for each independent experiment can be found in the supporting file, S2 Data, S3B and S3D Fig (C, F) *n* = 3. (A–D, F) Underlying numerical data and statistical analysis can be found in the supporting file, S2 Data, S3A–S3D and S3F Fig Values denote mean ± SD. *p < 0.05; ** *p* < 0.01; *** p <0.001.
(TIFF)

S4 Fig Metabolic drugs do not affect CD4^+^/CD8^+^ T cell ratio and CD25 expression. Representative FACS dot plots and histograms showing cell surface expression of CD4, CD8, and CD25 on activated T cells treated with Rapamycin (200 nM), 2-DG (1 mM) or Metformin (2 mM) as assessed by flow cytometry.
(TIFF)

S5 Fig Metabolic drugs do not affect T cell surface molecule phenotype and FSC/SSC profile. Representative FACS dot plots and histograms showing FSC/SSC profile and cell surface expression of CXCR3, CCR7, CD62L, and LFA-1 on activated T cells treated with Rapamycin (200nM), 2-DG (1 mM) or Metformin (2 mM) as assessed by flow cytometry.
(TIFF)

S6 Fig Cytokine expression profiles of Th0, Th1, Th2, and Th17 cell subsets. Relative mRNA expression levels of the cytokines *Ifn-γ*, *Tnf-β*, *Il-4*, *Il-5*, *Il-13*, and *Il-17* as assessed by qRT-PCR. mRNA levels of each cytokine expressed by untreated Th0 cells were set to 1. Data is representative of three independent experiments; the underlying numerical data and statistical analysis for each independent experiment can be found in the supporting file, S2 Data, S6 Fig.
(TIFF)

S7 Fig FACS dot plots of in vivo peritonitis model. (A, B) Representative peritoneal lavage FACS dot plots of activated CD4^+^ (A) and CD8^+^ (B) T cells of C57BL/6 mice injected i.p. with zymosan to induce peritonitis, and 5 d later treated with Slc5a12 specific antibody (5 μg/ml), phloretin (50 μM) or isotype control antibody, which correspond to the CD4^+^ and CD8^+^ T cells in the peritoneal lavage shown in Fig 6B. (C) Peritoneal lavage FACS dot plots of adoptively transferred CFSE-labeled activated CD4^+^ T cells, which are representative of the analyses shown in Fig 6C.
(TIFF)

S1 Table Demographical data of RA patients. (DOCX)

S2 Table List of primers used for qRT-PCR experiments. (XLSX)

## Abbreviations

2-DG: 2-deoxyglucose
2-NBDG: 2-(N-(7-Nitrobenz-2-oxa-1,3-diazol-4-yl)Amino)-2-Deoxyglucose
6-NBDG: 6-(N-(7-Nitrobenz-2-oxa-1,3-diazol-4-yl)Amino)-2-Deoxyglucose
CCL: chemokine (C-C motif) ligand
CFSE: carboxyfluorescein succinimidyl ester
CHC: α-cyano-4-hydroxycinnamate
CID: chronic inflammatory disease
CTL: cytotoxic T lymphocyte
CXCL: chemokine (C-X-C motif) ligand
DDAO: 7-Hydroxy-9H-(1,3-Dichloro-9,9-Dimethylacridin-2-One)
EC50: half maximal effective concentration
ECAR: extracellular acidification rate
Hk: hexokinase
IC50: half maximal inhibitory concentration
IFN-γ: interferon-gamma
IL: interleukin
Ki: inhibitor constant
Mct: monocarboxylate transporter
OA: osteoarthritis
PBS: phosphate buffered saline
Pfk: phosphofructokinase
PFKFB3: 6-phosphofructo-2-kinase/fructose-2,6-biphosphatase 3
Pk: pyruvate kinase
qRT-PCR: quantitative reverse transcription polymerase chain reaction
RA: rheumatoid arthritis
shRNA: short hairpin RNA
TCR: T-cell receptor
TGF-β: transforming growth factor-beta
