# Supplementary figures and images for: Lactate Regulates Metabolic and Pro-inflammatory Circuits in Control of T Cell Migration and Effector Functions
Source: PLoS Biol. 2015 Jul 16;13(7):e1002202. doi: 10.1371/journal.pbio.1002202 (PMC4504715; doi:10.1371/journal.pbio.1002202)

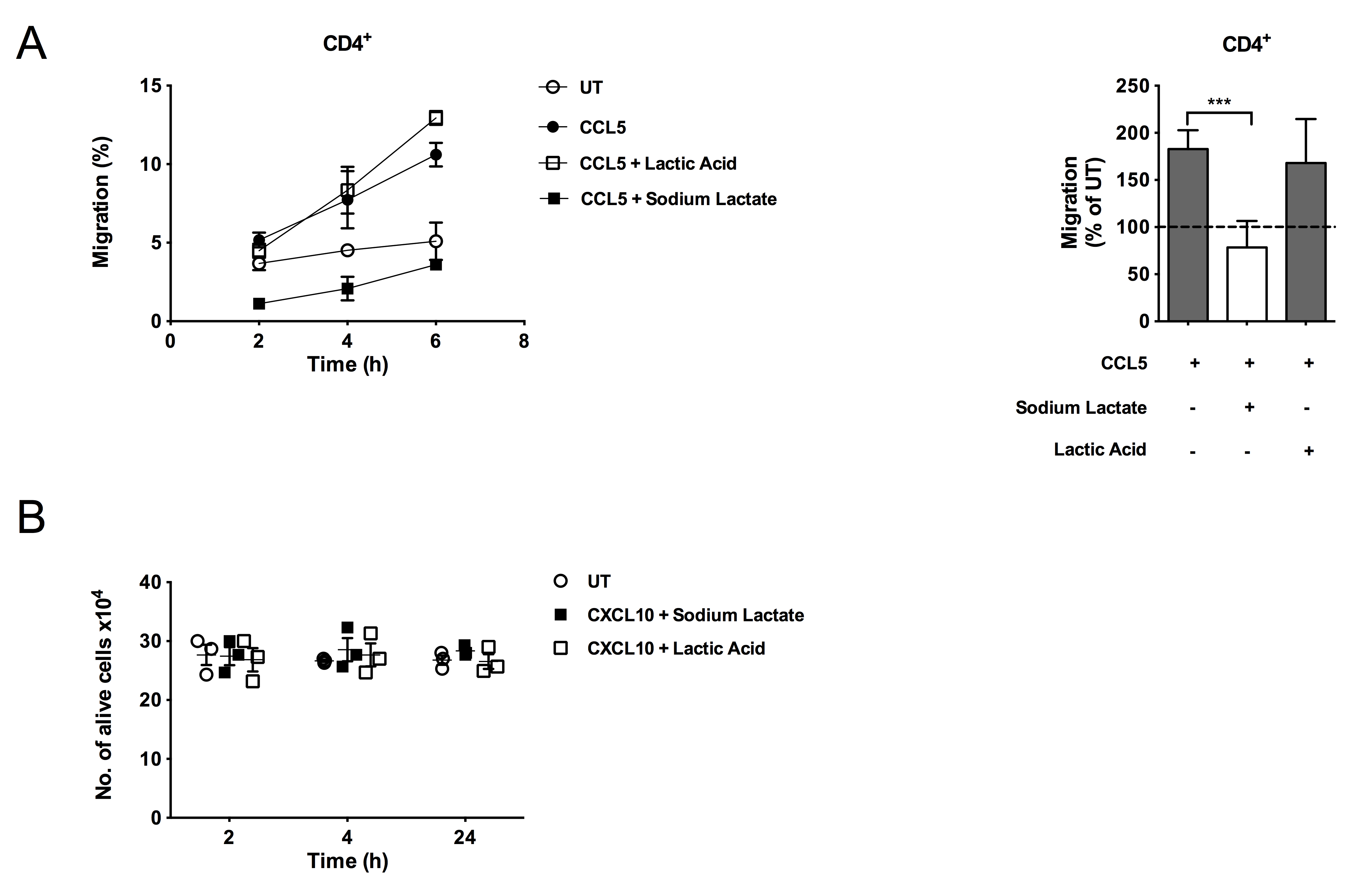

Supplement: S1 Fig — (A) In vitro chemotaxis of activated CD4+ T cells towards CCL5 (50 ng/ml) in the presence of lactic acid (10 mM) or sodium lactate (10 mM), shown as kinetic (left panel) and 4 h time point (right panel). (B) Total cell number of viable CD4+ T cells treated with CXCL10 in the presence of lactic acid (10 mM) or sodium lactate (10 mM). (A left panel) Data is representative of three independent experiments; the underlying numerical data and statistical analysis for each independent experiment can be found in the supporting file, S2 Data, S1A Fig (A right panel, B) n = 3. (A–B) Underlying numerical data and statistical analysis can be found in the supporting file, S2 Data, S1A–S1B Fig Values denote mean ± SD. ***p < 0.001. (TIFF) [file pbio.1002202.s003.tiff]

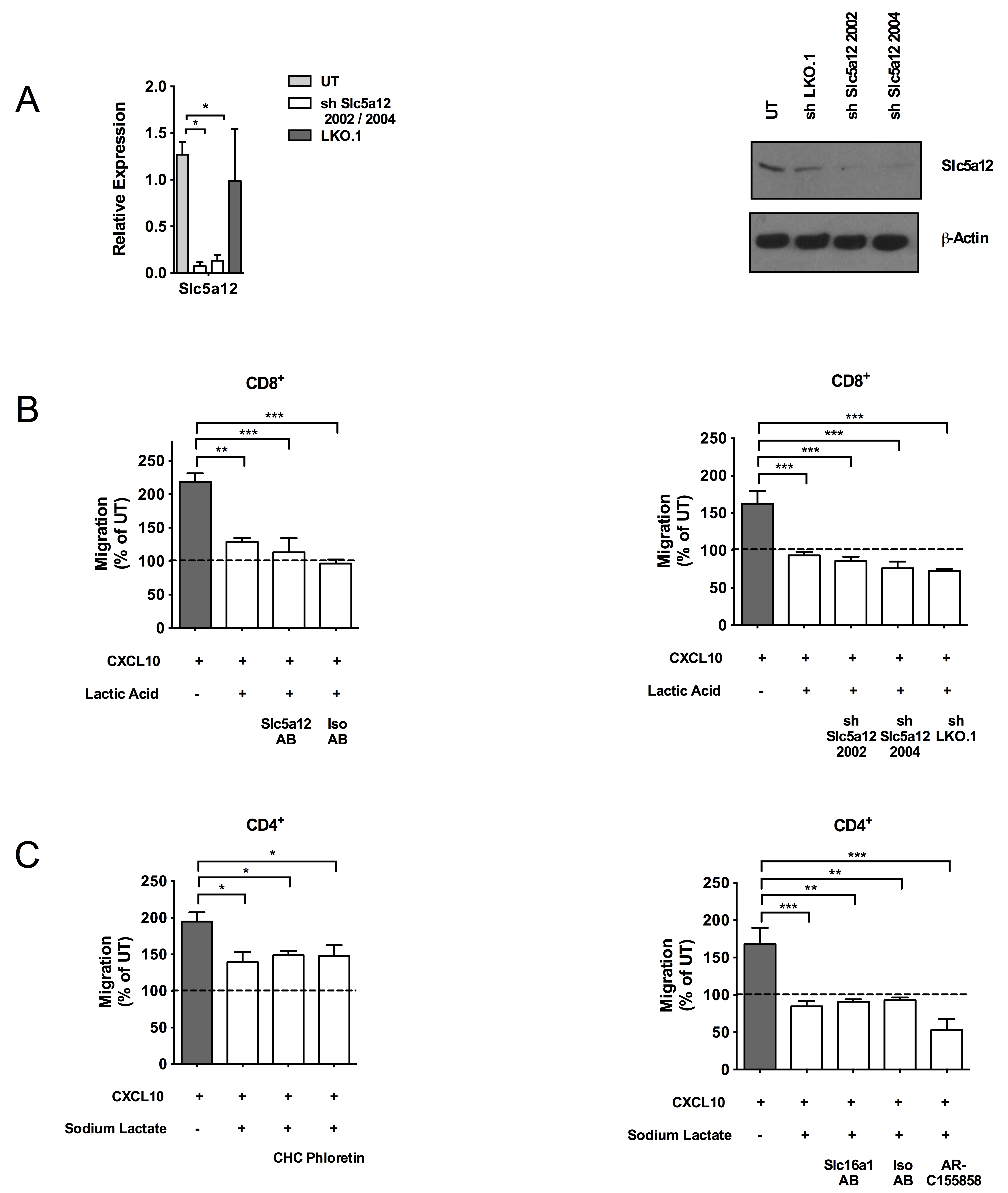

Supplement: S2 Fig — (A) Western blots and qRT-PCR with Slc5a12-specific primers and RNAs from activated CD4+ T cells expressing the shRNAs shown. (B, C) In vitro chemotaxis (4 h time point) of activated CD8+ T cells towards CXCL10 in the presence of lactic acid (10 mM) alone or in combination with an anti-Slc5a12 antibody (2.5 μg/ml) or an isotype control antibody (B left panel) and two specific shRNAs for Slc5a12 or a nonspecific shRNA (B right panel), and activated CD4+ T cells towards CXCL10 in the presence of sodium lactate alone or in combination with CHC (425 μM) or phloretin (25 μM) (C left panel) and an anti-Slc16a1 (2.5 μg/ml) or an isotype control antibody, or AR-C155858 (8 nM) (C right panel). (A) Data is representative of three independent experiments; the underlying numerical data and statistical analysis for each independent experiment can be found in the supporting file, S2 Data, S2A Fig (B–C) n = 3. (A–C) Underlying numerical data and statistical analysis can be found in the supporting file, S2 Data, S2A–S2C Fig Values denote mean ± SD.*p < 0.05; ** p < 0.01; ***p < 0.001. (TIFF) [file pbio.1002202.s004.tiff]

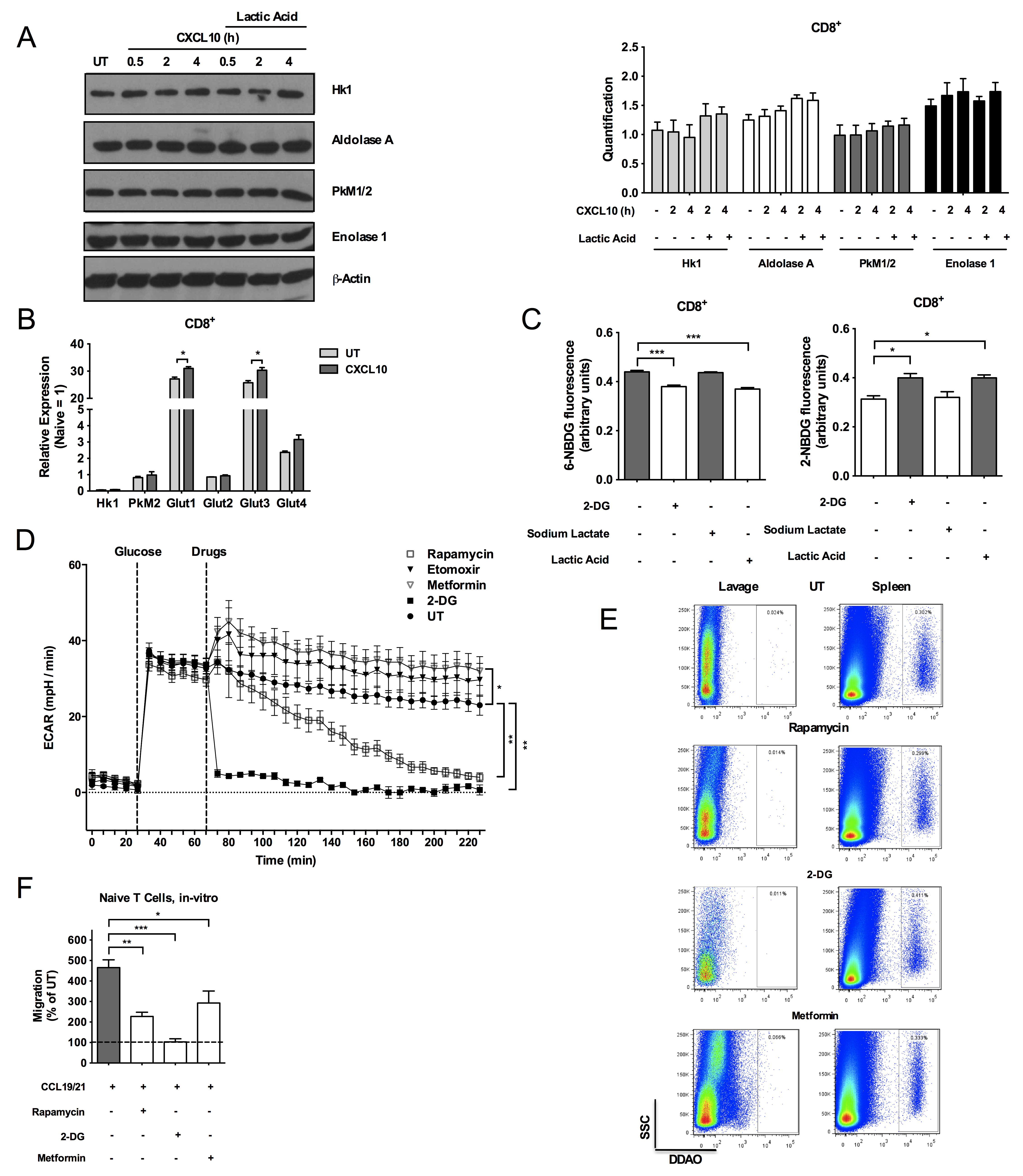

Supplement: S3 Fig — (A) Western blots with antibodies against Hk1, aldolase A, PkM1/2, enolase 1, and β-actin in activated CD8+ T cells treated with CXCL10 (1,000 ng/ml) or left untreated. Densitometric quantification of western blots denotes mean ± SD, n = 3 (with biological replicates run in duplicate). (B) Relative mRNA expression levels of Hk1, PkM2, and glucose transporters (Glut1, Glut2, Glut3, and Glut4) in activated CD8+ T cells 6 h post-treatment with CXCL10 (1,000 ng/ml) as assessed by qRT-PCR. mRNA levels in naive T cells were set to 1. (C) Measurements of glucose uptake and flux in activated CD8+ T cells pretreated with 2-DG, sodium lactate or lactic acid and then incubated with the fluorescent probes 6-NBDG or 2-NBDG. (D) ECAR trace of glycolytic activity expressed as mpH/min in activated CD4+ T cells treated with 2-DG (1 mM), Rapamycin (200 nM), Metformin (2 mM) or Etomoxir (100 μM). (E) Representative FACS dot plots of DDAO-labelled donor CD4+ T cells collected from the peritoneal lavage and spleen of recipient mice, which correspond to the relative enrichment in peritoneal lavage shown in Fig 3G. (F) In vitro chemotaxis (4 h time point) towards the chemokines CCL19/21 (200 ng/ml of each chemokine) of naïve T cells pretreated with Rapamycin (200 nM), 2-DG (1 mM) or Metformin (2 mM). (B, D) Data is representative of three (B) and two (D) independent experiments; the underlying numerical data and statistical analysis for each independent experiment can be found in the supporting file, S2 Data, S3B and S3D Fig (C, F) n = 3. (A–D, F) Underlying numerical data and statistical analysis can be found in the supporting file, S2 Data, S3A–S3D and S3F Fig Values denote mean ± SD. *p < 0.05; ** p < 0.01; *** p <0.001. (TIFF) [file pbio.1002202.s005.tiff]

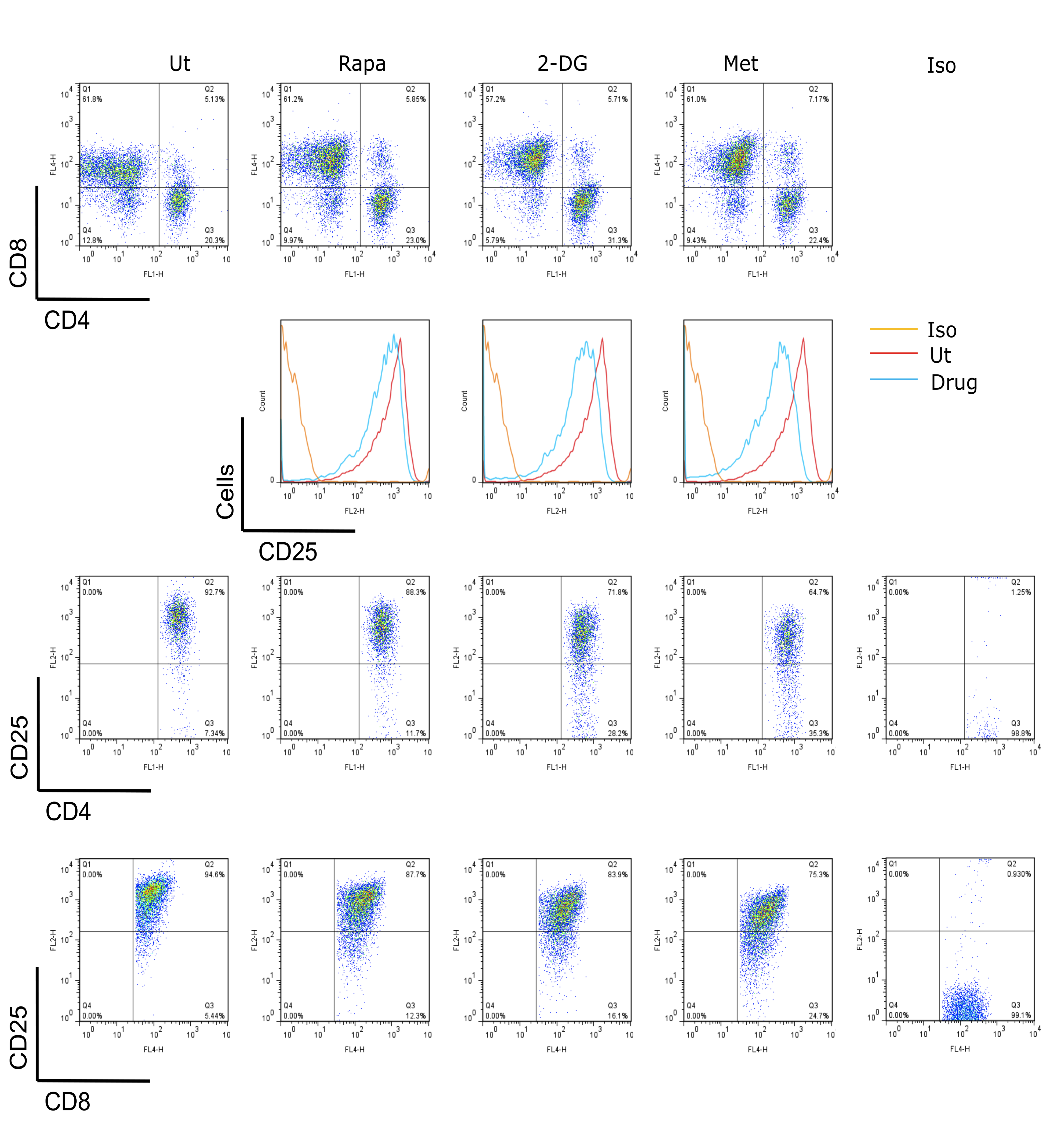

Supplement: S4 Fig — Representative FACS dot plots and histograms showing cell surface expression of CD4, CD8, and CD25 on activated T cells treated with Rapamycin (200 nM), 2-DG (1 mM) or Metformin (2 mM) as assessed by flow cytometry. (TIFF) [file pbio.1002202.s006.tiff]

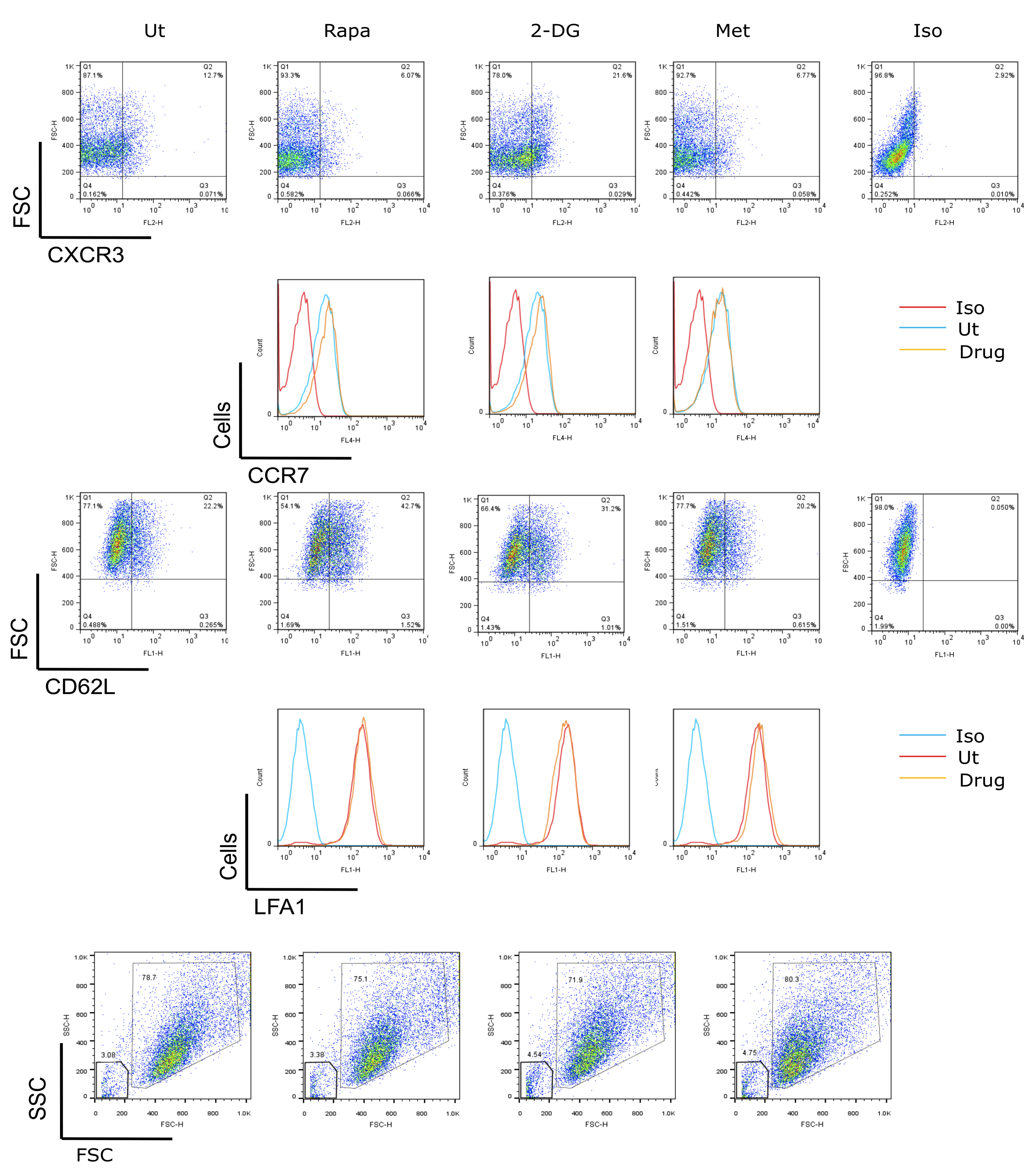

Supplement: S5 Fig — Representative FACS dot plots and histograms showing FSC/SSC profile and cell surface expression of CXCR3, CCR7, CD62L, and LFA-1 on activated T cells treated with Rapamycin (200nM), 2-DG (1 mM) or Metformin (2 mM) as assessed by flow cytometry. (TIFF) [file pbio.1002202.s007.tiff]

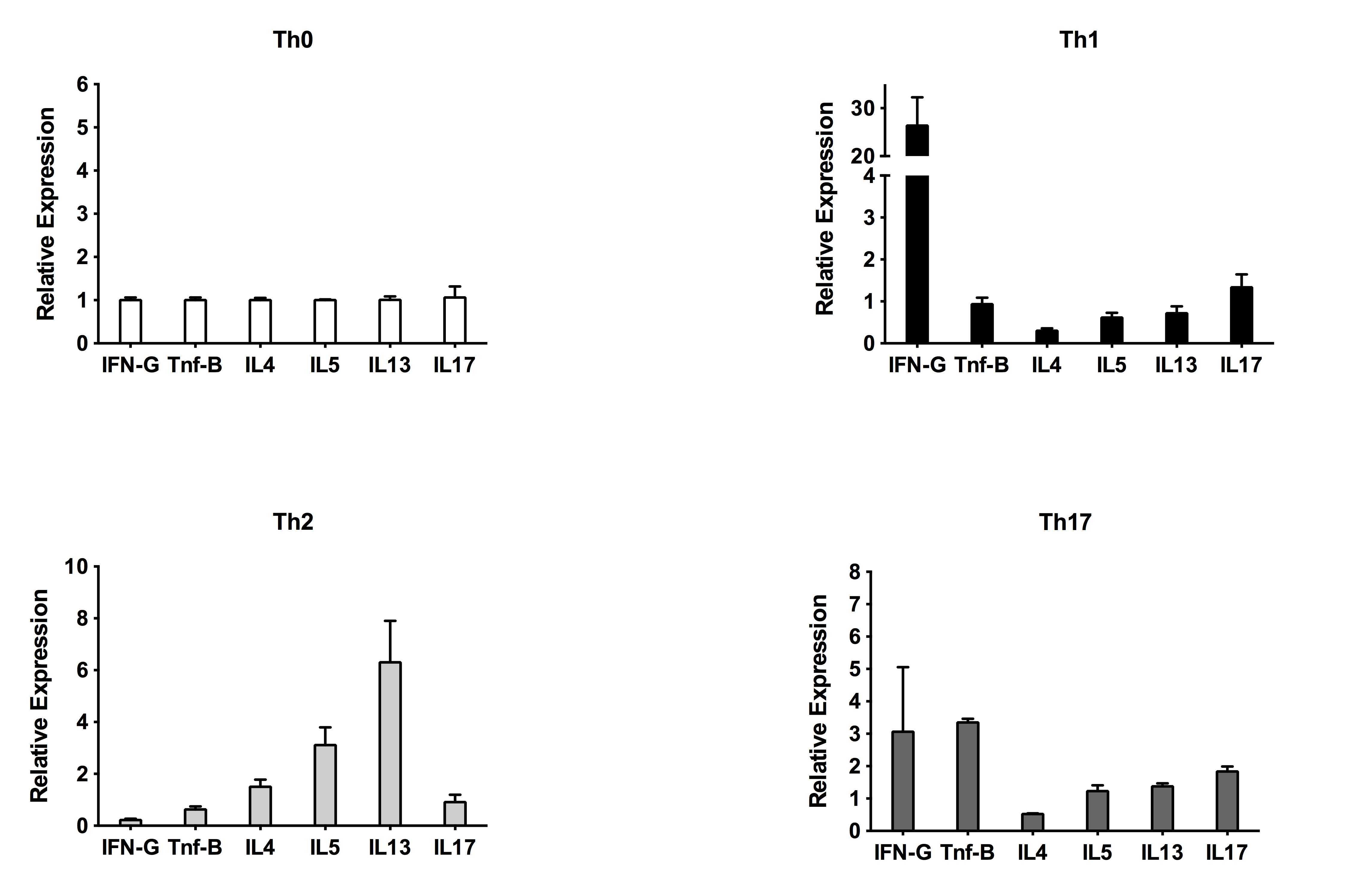

Supplement: S6 Fig — Relative mRNA expression levels of the cytokines Ifn-γ, Tnf-β, Il-4, Il-5, Il-13, and Il-17 as assessed by qRT-PCR. mRNA levels of each cytokine expressed by untreated Th0 cells were set to 1. Data is representative of three independent experiments; the underlying numerical data and statistical analysis for each independent experiment can be found in the supporting file, S2 Data, S6 Fig. (TIFF) [file pbio.1002202.s008.tiff]

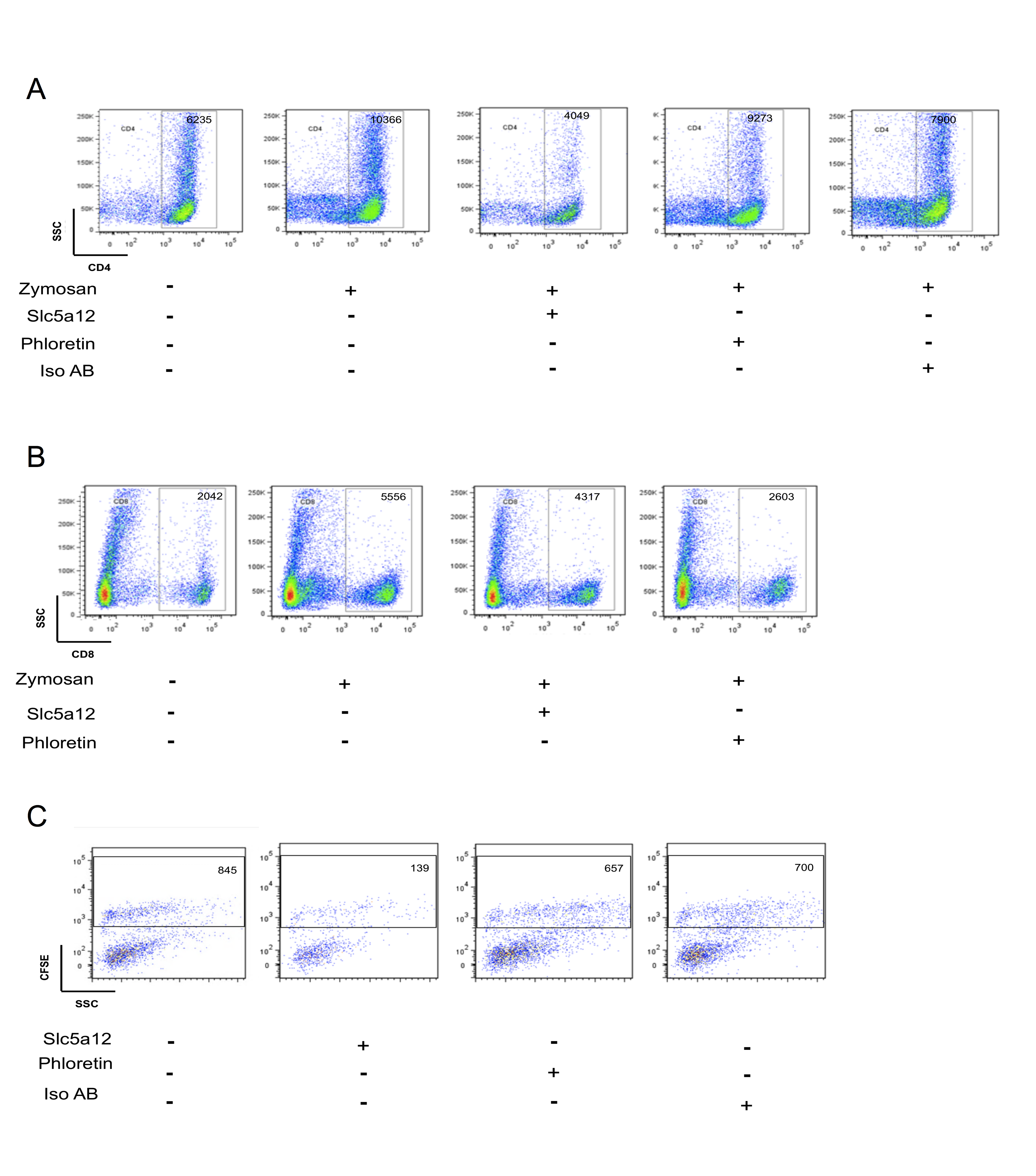

Supplement: S7 Fig — (A, B) Representative peritoneal lavage FACS dot plots of activated CD4+ (A) and CD8+ (B) T cells of C57BL/6 mice injected i.p. with zymosan to induce peritonitis, and 5 d later treated with Slc5a12 specific antibody (5 μg/ml), phloretin (50 μM) or isotype control antibody, which correspond to the CD4+ and CD8+ T cells in the peritoneal lavage shown in Fig 6B. (C) Peritoneal lavage FACS dot plots of adoptively transferred CFSE-labeled activated CD4+ T cells, which are representative of the analyses shown in Fig 6C. (TIFF) [file pbio.1002202.s009.tiff]
