# Supplementary material for: Lactate Regulates Metabolic and Pro-inflammatory Circuits in Control of T Cell Migration and Effector Functions
Source: PLoS Biol. 2015 Jul 16;13(7):e1002202. doi: 10.1371/journal.pbio.1002202 (PMC4504715; doi:10.1371/journal.pbio.1002202)
Supplement: S1 Table — (DOCX) [file pbio.1002202.s010.docx]

| Parameter | Study Population  *(n=16)* |
| --- | --- |
| Age (range) | 46-76 |
| Gender (%) |  |
| *Female* | 80 |
| *Male* | 20 |
| Site (%) |  |
| *Large joint* | 68 |
| *Small joint* | 32 |
| Erosive (%) | 63 |
| Treatment (%) |  |
| *DMARDs* | 81 |
| *Steroids* | 15 |
| *Biologics* | 61 |
| RF^+^ and/or CCP^+^ (%) | 67 |
